# LncRNA HOTAIR regulates lipopolysaccharide-induced cytokine expression and inflammatory response in macrophages

**DOI:** 10.1038/s41598-018-33722-2

**Published:** 2018-10-23

**Authors:** Monira Obaid, S. M. Nashir Udden, Paromita Deb, Nadine Shihabeddin, Md. Hasan Zaki, Subhrangsu S. Mandal

**Affiliations:** 1 Department of Chemistry and Biochemistry, The University of Texas at Arlington, Arlington, Texas 76019 USA; 2 Department of Pathology, The University of Texas Southwestern Medical Center, Dallas, Texas 75390 USA

## Abstract

Long noncoding RNAs (lncRNAs) are emerging as major regulators of a variety of cell signaling processes. Many lncRNAs are expressed in immune cells and appear to play critical roles in the regulation of immune response. Here, we have investigated the potential role of a well-known lncRNA, HOTAIR, in inflammatory and immune response. Our studies demonstrate that HOTAIR expression is induced in immune cells (macrophages) upon treatment with lipopolysaccharide (LPS). Knockdown of HOTAIR reduces NF-κB-mediated inflammatory gene and cytokine expression in macrophages. Inhibition of NF-κB resulted in down-regulation of LPS-induced expression of HOTAIR as well as IL-6 and iNOS expression. We further demonstrated that HOTAIR regulates activation of NF-κB and its target genes (IL-6 and iNOS) expression via facilitating the degradation of IκBα. HOTAIR knockdown reduces the expression of NF-κB target gene expression via inhibiting the recruitment of NF-κB and associated cofactors at the target gene promoters. Taken together, our findings suggest that HOTAIR is a critical player in NF-κB activation in macrophages suggesting its potential functions in inflammatory and immune response.

## Introduction

The mammalian immune system orchestrates innate and adaptive immune responses that are complex biochemical processes regulated by various protein and lipid mediators such as pattern recognition receptors, cytokines, chemokines, hormones, growth factors, and prostaglandins, etc^1–4^. Toll-like receptors (TLRs) play central roles in the innate reaction to bacterial infection and in immune responses against pathogens^5–8^. In particular, Toll-like receptor 4 (TLR4) is activated by endotoxin lipopolysaccharide (LPS) present in the outer membrane of Gram-negative bacteria^9–13^. TLR4 activation triggers a series of downstream signaling cascades including NF-κB activation^14–19^ and induction of cytokines, chemokines, and pro-inflammatory genes^6,20–24^. Increasing evidences suggest that noncoding RNAs (ncRNAs) are integral components of variety of cellular and physiological signaling processes including in immune response^25–29^. Notably ncRNAs are recently discovered classes of transcripts, which are coded by the genome, but remains mostly untranslated into proteins^30–34^. Noncoding RNAs, which are longer than 200 nucleotides, are classified as long-noncoding RNAs (lncRNAs)^35–37^. It is estimated that human genome encodes more than 28000 lncRNAs, though their functions and detailed characterization are yet to be revealed^25,36,38^. Examples of lncRNAs implicated in immune response include lincRNA-Cox2, linc1992/THRIL, nc-1L7R, NeST RNA, NEAT1 and others^38–41^. LincRNA-Cox2 regulates IL-6 expression, linc1992/THRIL regulates expression of IL-8, CXCL10, CCL1, and CSF1^39^, nc-1L7R knockdown diminishes IL-6 and IL-8 mRNA levels^40^, NeST RNA induces secretion of IFNγ in CD8^+^ T cells^41^, NEAT1 causes transcriptional activation of IL-8^39^ and so on. Here we explored potential roles of a well-known lncRNA called HOTAIR (HOX transcript antisense intergenic RNA) in cytokine regulation and immune response. HOTAIR is a 2.2 kb long lncRNA, an antisense transcript and is associated with gene repression^42–44^. HOTAIR interacts with gene silencing and chromatin modifying factors such as polycomb repressive complex 2 (PRC2)^25^ and lysine specific demethylase 1 (LSD1, a histone H3K4-demethylase) complexes^42,45^. EZH2 (Enhancer of zeste homolog 2) is a H3K27-methylase which is an interacting component of PRC2^46–48^. H3K27-methylation by EZH2 and H3K4-demethylation by LSD1 are crucial to gene silencing^42,49,50^. HOTAIR facilitates recruitment of PRC2 and LSD1 multi-protein complexes at the target genes promoters, which induce H3K27-methylation and H3K4-demethylaytion respectively and contribute to gene silencing^45,51^. HOTAIR upregulation is associated with a variety of cancers^37,52^. Beyond gene repression, HOTAIR is also found to be involved in protein degradation associated with neuronal function and diseases^53,54^. Previous studies from our laboratory showed that HOTAIR is required for the viability of breast cancer cells and its expression is regulated by estradiol and hypoxia^55–57^. Here, we demonstrate that HOTAIR is a critical player in LPS-induced cytokine expression, immune, and inflammatory response in macrophages.

## Materials and Methods

### Cell culture and treatment with Lipopolysaccharide (LPS)

Mouse macrophage RAW264.7 cells were obtained from American Type Cell Culture Collection, and cultured in Dulbecco’s modified Eagle’s medium (DMEM; Sigma-Aldrich, St. Louis, MO) supplemented with 10% heat-inactivated FBS (Fetal bovine serum), 2 mM L-glutamine, 100 units/mL penicillin and 0.1 mg/mL streptomycin in a humidified incubator with 5% CO2 and 95% air at 37 °C^58^. 2 × 10^6^ cells were seeded in 60 mm cell culture plates and after overnight culture cells were treated with ultrapure *E.coli*-derived LPS (Invivogen) at 1.0 μg/mL concentration for different time periods^58^. Notably, this concentration of LPS has been widely used in various other laboratories to induce immune and pro-inflammatory response in macrophages^58,59^. Cells were harvested for the preparation of RNA and protein analysis.

### RNA extraction, cDNA synthesis and real time PCR

Total RNA was extracted using TRIzol (Invitrogen) according to the manufacturer’s instructions. Briefly RAW264.7 cells treated with various conditions were harvested using TRIzol, mixed with chloroform and centrifuged at 12000 rpm for 15 min. The aqueous layer was mixed with equal volume of 100% ethanol and centrifuged at 12000 rpm for 10 min. The pellet was washed with 70% ethanol, and RNA was finally dissolved in 30–50 μL of RNase-free water (Sigma) and quantified using a Nanodrop spectrophotometer^58^. 1 μg of the isolated RNA was reverse transcribed into cDNA using iScript RT-supermix (Bio-Rad). For semi quantitative PCR the cDNA was PCR-amplified by using Taq DNA Polymerase (Genscript) as described earlier^60^ and primers are listed in Table 1. Real-time PCR was performed using iTaq Universal SYBR Green Supermix (Bio-Rad), with gene specific PCR primers as listed in Table 1, in CFX96 real-time detection system (Bio-Rad). Each experiment was repeated three times with three parallel replicates each time. Expression data were normalized to GAPDH and expressed as 2^−ΔCt ^^61,62^.

**Table 1:** Sequences of primers and antisense oligonucleotides.

Primers	Forward (5′-3′)	Reverse (5′-3′)
**PCR Primers**		
HOTAIR	TCCAGATGGAAGGAACTCCAGACA	ATAGATGTGCGTGGTCAGATCGCT
IL-6	CAAGAAAGACAAAGCCAGAGTC	GAAATTGGGGTAGGAAGGAC
iNOS	TGTGACACACAGCGCTACAACA	GAAACTATGGAGCACAGCCACAT
GAPDH	ACCCAGAAGACTGTGGATGG	CACATTGGGGGTAGGAACAC
FgB	CTATGGCTGCTGCTGCTATTG	GGCTCTTCCTTTCTCCTGTCAAC
Mt1	AATGTGCCCAGGGCTGTGT	GCTGGGTTGGTCCGATACTATT
TNFα	TCCCAGGTTCTCTTCAAGGGA	GGTGAGGAGCACGTAGTCGG
MIP-1B	CCTGACCAAAAGAGGCAGAC	GAGGAGGCCTCTCCTGAAGT
**ChIP PCR Primers**		
IL-6 promoter^a^	GCACACTTTCCCCTTCCTAGTT	AGACTCATGGGAAAATCCCACATT
iNOS-promoter^a^	GTGTACCTCAGACAAGGGCA	AAGCATTCACACATGGCATGGA
**Antisense Oligonucleotides**		
HOTAIR Antisense	5′-C*C*T*T*C*C*T*T*C*C*G*C*T*C*T*T*A*C*T*C*T-3′	
Scramble Antisense	5′-C*C*T*C*T*T*C*T*G*T*C*T*C*T*T*C*C*C*G*C*T-3′	

^a^ChIP PCR primers are flanked around the NF-κB binding site.

*All phosphorothioate linkages instead of regular phosphodiester bonds.

### Western blot analyses

The cells were washed in ice-cold PBS and then lysed in cell lysis buffer comprising 50 mM Tris–HCl (pH 8.0), 150 mM NaCl, 5 mM EDTA, 1% Igepal CA-630, 0.5% Na-deoxycholate, 2 mM Na_3_VO_4_, and complete protease inhibitor cocktail and phosphatase inhibitor cocktail (Roche)^63^. The resulting cell lysates were centrifuged for 10 min at 13,000 rpm at 4 °C, and the protein concentrations in the supernatants were determined using a BCA protein assay kit (Pierce). 20 μg proteins were loaded onto 10% SDS-PAGE gels, followed by electro-transfer onto PVDF-membrane (Immobilon-P, Millipore). The membranes were blocked in 1× TBST (0.1% Tween-20, 20 mM Tris–Cl (pH 8.0), and 150 mM NaCl) containing 5% skimmed milk and then incubated with the primary antibodies against IκBα (1:1000 dilution, 4814S, Cell Signaling), Phospho-IκBα (1:1000 dilution, 2859S, Cell Signaling), Phospho-p65 (NF-κB subunit, 1:1000 dilution, 3033S, Cell Signaling), IL-6 (1:1000 dilution, 12912S, Cell Signaling), iNOS (1:1000 dilution, 13120S, Cell Signaling), and β-actin (1:1000 dilution, A2066, Sigma) overnight at 4 °C. Membranes were washed 3 times (1xTBST), incubated with horseradish peroxidase-conjugated secondary antibodies for 1 h at room temperature and then washed 3 times (1 × TBST). Finally, immunoreactive proteins were detected using ECL-super signal west femto substrate reagent (Thermo Scientific)^63^. The amount has been quantified by ImageLab5.2.1software.

### Chromatin Immunoprecipitation (ChIP) assay

The ChIP assay was performed as described earlier^55,60^. Briefly, the cells were cross-linked with 1% formaldehyde for 10 min at 37 °C, washed twice in ice-cold PBS and harvested using SDS lysis buffer (1% SDS, 10 mM EDTA, 50 mM Tris. HCl, pH 8.1) supplemented with complete protease inhibitor (Roche). Cells were subjected to sonication to shear the chromatin (∼200–300 bp range). The fragmented chromatin was pre-cleared with protein G agarose beads (16-266, EMD Millipore) and subjected to immunoprecipitation using antibodies specific to CBP (A22, Santa Cruz Biotechnology, Sc369), p300 (N15, Santa Cruz Biotechnology, Sc584), Phospho-p65 (3033, Cell Signaling), histone H3K4-trimethyl (07-473, EMD-Millipore), histone acetylation (06-599, EMD-Millipore), RNA Pol II (8WG16, Abcam), and β-actin (A2066, Sigma). Immunoprecipitated chromatin was washed, de-crosslinked and deproteinized at 65 °C in presence of 5 M NaCl followed by incubation with proteinase K (Sigma) at 45 °C for 1 h. Purified ChIP DNA fragments were analyzed by semi-quantitative PCR and real-time PCR using primers spanning NF-κB binding sites present in the IL-6 and iNOS promoters (Table 1)^64–66^.

### Antisense-mediated knockdown of HOTAIR

For the antisense transfection, RAW 264.7 cells were grown up to 60% confluency in 60 mm culture plates and transfected with HOTAIR-antisense (HOTAIR-AS) and scramble-AS (no homology to HOTAIR)^55,56^ independently (Table 1) using GenMute transfection reagent (SL100568, SignaGen Laboratories) according to the manufacturer’s protocol. Prior to transfection, a cocktail of transfection reagent and antisense oligonucleotides was made as follows. Initially, 12 μL (12 μg) of GenMute reagent was mixed with 300 μL DMEM (without FBS and antibiotics) in an eppendorf tube. In a separate eppendorf, antisense oligonucleotide was mixed with 100 μL DMEM (without supplements), then mixed with diluted GenMute reagents and allowed to stand for 30 min in dark. In the meantime, cells were washed twice with supplement-free DMEM, and then 1.7 mL of supplement-free DMEM was added. Finally, antisense transfection reagents cocktail was applied, mixed gently and incubated for 48 h. Cells were then stimulated with LPS (1 μg/mL) for specified time period and then harvested for RNA/protein extraction or for ChIP assays.

### SiRNA-mediated knockdown of HOTAIR

For the siRNA transfection, RAW 264.7 cells were grown up to 60% confluency in 60 mm culture plates and transfected with HOTAIR-siRNA, a pool of 4 different siRNA constructs (SI05685183, SI05685190, SI05685197, and SI05685204 Qiagen)^67^ and scramble siRNA (1027310 Qiagen) independently using GenMute transfection reagent (SL100568, SignaGen Laboratories) according to the manufacturer’s protocol. Prior to transfection, a cocktail of transfection reagent and antisense oligonucleotide was made as follows. Initially, 12 μL (12 μg) of GenMute reagent was mixed with 300 μL DMEM (without FBS and antibiotics) in an eppendorf tube. In a separate eppendorf, siRNA was mixed with 100 μL DMEM (without supplements). Then the diluted siRNA solution was mixed with diluted GenMute reagents and allowed to stand for 30 min in the dark. Cells were washed twice with supplement-free DMEM, 1.7 mL of supplement-free DMEM was added and finally, siRNA transfection reagents cocktail was applied to the cell plates, mixed gently and incubated for 48 h. Cells were then stimulated with LPS (1 μg/mL) for specified time period and harvested for RNA/protein extraction.

### NF-κB inhibition assay

RAW264.7 macrophages (2 × 10^6^) were seeded in 60 mm cell culture plates. After overnight incubation cells were initially treated with IKKβ inhibitor (25 μM, SC-514, Sigma)^68^ for 1 h to inhibit NF-kB signaling pathway and then cells were treated with LPS (1 μg/mL) and incubated for additional period of time 4 h. Cells were harvested, total RNA was isolated using TRIzol reagent, reverse transcribed to cDNA and analyzed by qPCR for the expression of HOTAIR, IL-6 and iNOS. GAPDH was used as control. Protein was also extracted (SC-514 1 h and additional1 h LPS treatment) after cell harvesting for Western blot.

### Proteasomal inhibition assay

RAW264.7 macrophages (2 × 10^6^) were seeded in 60 mm cell culture plates. After overnight incubation cells were initially treated with or without HOTAIR-AS for 48 h. The cells were then treated with MG132 (Sigma) (10 μM) for 2 h followed by LPS (1 μg/mL) and incubated for additional 1 h and followed the procedure for immunofluorescence microscopy analysis^69^.

### Enzyme linked immunosorbent assay (ELISA)

HOTAIR was silenced in RAW264.7 macrophages by using HOTAIR-AS and HOTAIR-siRNA, a pool of 4 different siRNA constructs (SI05685183, SI05685190, SI05685197, and SI05685204 Qiagen) by the use of GenMute siRNA transfection reagent (SL100568, SignaGen Laboratories) according to the manufacturer’s protocol. 48 h following transfection RAW264.7 macrophages were stimulated with LPS for 12 h or kept untreated. Culture media were collected and amount of IL-6 secreted in culture media were measured using ELISA kits (DY406-05 R&D Systems) according to manufacturer’s instruction^58^.

### Immunofluorescence microscopy analysis

For immunofluorescence staining of macrophages, cells were seeded on cover slips and fixed in 4% paraformaldehyde (PFA) for 15 min at room temperature, washed with 1X PBS (3 times for 5 min each) and blocked with 1X PBS containing 5% goat normal serum and 0.3% Triton-X100 for 1 h. The cells were then incubated with primary antibodies (rabbit anti-P-p65, 1:200, 3033, CST; and mouse anti-IκBα (L35A5, 1:400, 4814, CST) overnight at 4 °C. After that the cells were washed 3 times with PBS followed by incubation with anti-rabbit Alexa Fluor 564 (Invitrogen) and anti-mouse Alexa Fluor 488 (Invitrogen), conjugated secondary antibodies for 1 h at RT. Finally the cells were washed 3 times with PBS and mounted with mounting media containing DAPI. Images were taken by fluorescence microscope (Nikon ECLIPSE TE2000-U). The amount has been quantified by the National Institutes of Health (NIH) ImageJ software.

### Mice

Wild-type (C57BL6/J) mice were purchased from Jackson Laboratory. All mice are maintained in a specific pathogen free (SPF) facility at UT Southwestern Medical center. All studies were approved by the Institutional Animal Care and Use Committee (IACUC) and were conducted in accordance with the IACUC guidelines and the National Institutes of Health Guide for the Care and Use of Laboratory Animals.

### Isolation and culture of primary macrophage (bone marrow derived macrophage, BMDM)

BMDM was isolated from mice bones and cultured as described earlier^58,70^. For the isolation of BMDMs, femur and tibia were collected from mouse legs. Using 25G needle bone marrows were flushed out with Iscove’s Modified Dulbecco’s Medium (IMDM), (12440061, Life technologies) and processed for single cell suspension by passing through 22G needle two times. The suspension was centrifuged at 1000 RPM for 5 min. The pellet was re-suspended with BMDM culture media (L-cell-conditioned IMDM medium supplemented with 15% L929 supernatant, 10% FBS, 1% nonessential amino acid, and 1% penicillin-streptomycin) followed by seeding in three 150 mm culture dishes and cultured for 6 days to differentiate into macrophages, while at day 3, 10 ml fresh BMDM culture media was added into each plate. After day 6, the culture plate was washed with ice cold PBS and cells were gently scraped with ice cold PBS. The BMDM was centrifuged at 1000 rpm for 5 min and re-suspended into BMDM media. The BMDM was counted and seeded in 6-well (2.5 × 10^6^/well) cell culture plates. After overnight incubation the BMDM was treated with LPS or HOTAIR antisense oligonucleotides and processed for further experiments.

### Statistical Analysis

Each experiment was done in two or three replicates, and then cells were pooled (and treated as one sample), subjected to RNA extraction, RT-PCR, and ChIP analysis, and each experiment was repeated at least three times (n = 3). The real-time PCR analysis of such samples were done in three replicate reactions and repeated in all three independent experiments (n = 3). Data are presented as means ± SD (as stated in the figure legends). Statistical significance was determined by unpaired Student’s t test (GraphPad Prism 6), and P ≤ 0.05 was considered statistically significant.

## Results

### LncRNA HOTAIR expression is induced by Lipopolysaccharide in macrophages

**Figure 1:**
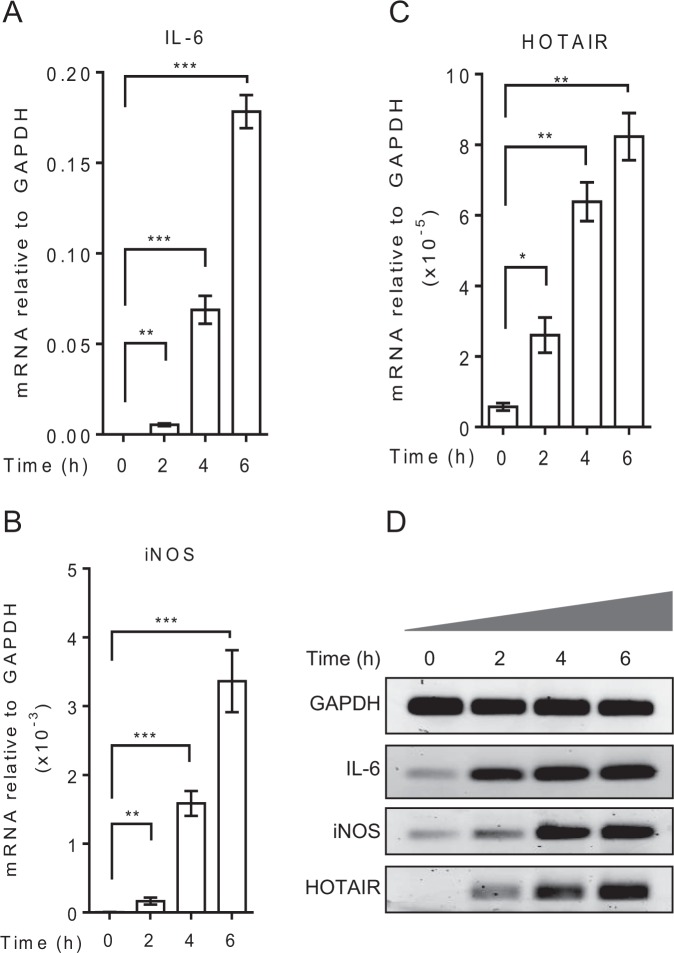
LPS induces HOTAIR expression in macrophages. RAW264.7 cells were treated with LPS (1 μg/mL) for varying period of time, total RNA was isolated, reverse transcribed to cDNA and analyzed by qPCR for expression of IL-6 (**A**) iNOS (**B**) and HOTAIR (**C**). cDNA was also analyzed by semi-quantitative PCR and agarose gel (**D**). Each experiment was repeated at least with three parallel replicates. GAPDH was used as loading control. Data represent mean ± SD (n = 3); *p < 0.05, **p < 0.001, ***p < 0.0001.

To investigate the roles of lncRNA in immune response, initially we explored potential involvement of lncRNA HOTAIR. As HOTAIR is well-known as a repressor^49,50,71,72^, initially we hypothesized that HOTAIR may be involved in repression of cytokine and inflammatory gene expression. To test this hypothesis, we treated macrophage cells (RAW264.7) with LPS and analyzed its impacts on the expression of HOTAIR along with well-known cytokines (e.g. interleukin-6, IL-6) and pro-inflammatory genes (e.g. inducible nitric oxide synthase, iNOS)^73,74^. RNA from the LPS-treated macrophages were analyzed by RT-qPCR. As expected, LPS-treatment induced the expression of IL-6 and iNOS in time-dependent manner (Fig. 1A,B). The expression of IL-6 was induced by 522, 6722 and 17321 folds at 2, 4 and 6 h post LPS-treatment, respectively (Figs 1A, S1A shows the fold change). Similarly, the expression of iNOS was also induced by 31, 292 and 621 folds at 2, 4 and 6 h respectively upon treatment with LPS (Figs 1B and S1A). Notably, along with IL-6 and iNOS, we also examined the LPS-induced expression of additional cytokines and pro-inflammatory genes, such as tumor necrosis factor α (TNFα), macrophage inflammatory protein-1β (MIP-1β), beta-fibrinogen (FgB) and metallothionein1 (Mt1) (Fig. S1B)^75,76^. TNFα and MIP-1β were significantly induced by LPS-treatment, while LPS-induced expressions of FgB and Mt1 were relatively low (Fig. S1B). Interestingly, along with IL-6, iNOS and other cytokines and inflammatory genes, lncRNA HOTAIR expression was also induced upon treatment with LPS (5, 11 and 14 folds at 2, 4 and 6 h, respectively) (Figs 1C and S1A). Notably, LPS-dependent induction of IL-6 and iNOS is much higher compared to HOTAIR expression, though the LPS-induced HOTAIR expression is significant. The cDNA was also analyzed by semi-quantitative PCR and products were analyzed on agarose gel showing the LPS-dependent induction of IL-6, iNOS and HOTAIR (Figs 1D and S1C). Overall, these observations demonstrated that along with IL-6, iNOS, lncRNA HOTAIR expression is induced upon stimulation with LPS and it may be associated with inflammatory and immune response in macrophages.

### Inhibition of NF-κB downregulates LPS-induced HOTAIR expression

**Figure 2:**
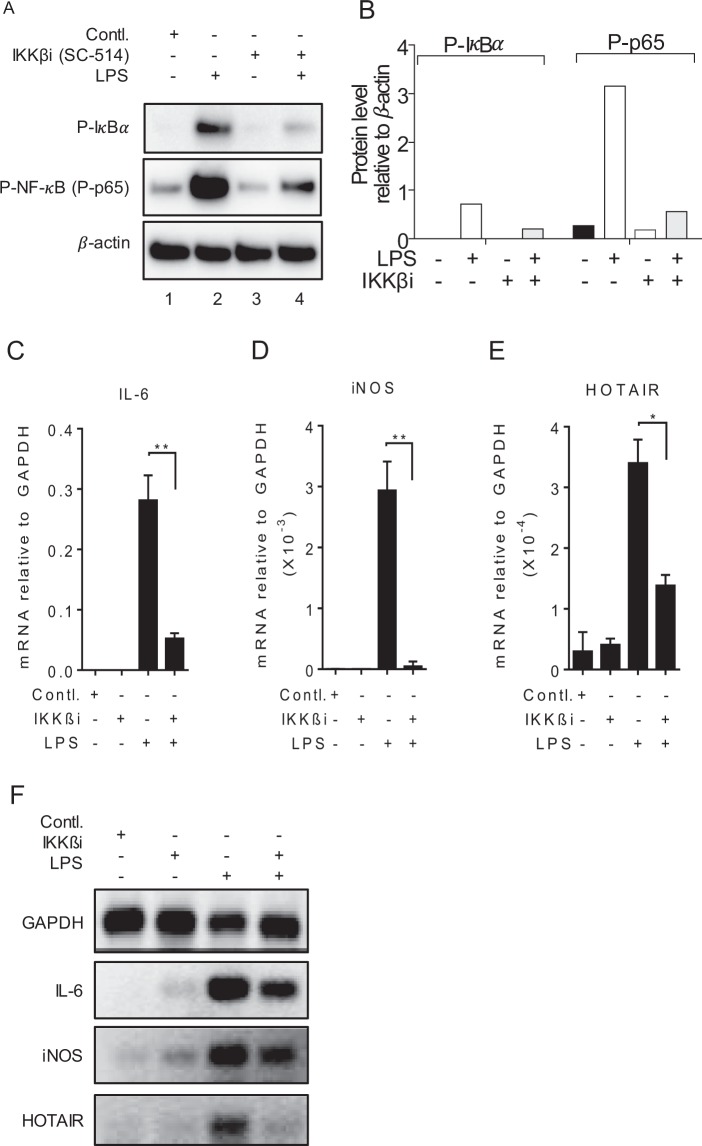
Inhibition of NF-κB downregulates LPS-induced HOTAIR expression in macrophages. RAW264.7 cells were initially treated with IKKβ-inhibitor SC-514 (for 1 h) and then treated with LPS for additional 1 h for protein and 4 h for RNA. Proteins were analyzed by Western blotting using antibody against phospho-IκBα, phospho-p65 (NF-κB subunit) and β-actin (loading control) (panel A, quantifications using ImageLab5.2.1software is shown in panel B). RNA was isolated and expressions of IL-6 (**C**) iNOS (**D**) and HOTAIR (**E**) were measured by RT-qPCR. cDNA was also analyzed by semi-quantitative PCR and agarose gel (**F**). GAPDH was used as control for PCR experiments. Data represent mean ± SD (n = 3); *p < 0.05, **p < 0.001.

The activation of transcription factor NF-κB is well-known to be associated with LPS-induced cytokine expression (including IL-6 and iNOS) and inflammatory response^77,78^. To investigate if NF-κB activation is associated with LPS-induced HOTAIR expression, we treated macrophages with an inhibitor of IKKβ (SC-514)^79^ and analyzed its impacts on LPS-induced HOTAIR, IL-6 and iNOS expressions. Notably, IKKβ is a kinase which phosphorylates IκBα allowing its poly-ubiquitination, proteasomal degradation, and hence NF-κB activation^6,77,80,81^. Thus, inhibition of IKKβ (by SC-514) results in deactivation of NF-κB^6,77^. Briefly, RAW264.7 cells were treated with IKKβ-inhibitor SC-514 (25 µM, 1 h) and stimulated with LPS. The concentration of the inhibitor (SC-514) used is chosen based on previous literature^68^. Proteins from the control and SC-514 treated cells were analyzed by Western blot to detect the levels of phospho-IκBα and phospho-p65^80,81^. As expected SC-514 treatment resulted in a decrease in phospho-IκBα and phospho-p65 (NF-κB submit) levels, indicating the effective inhibition of IKK*β* kinase activity and consequent decrease in NF-κB activation (Fig. 2A, quantifications in panel 2B, Supplementary Fig. S2A). The RNA from the control and SC-514 treated cells were also then analyzed by RT-qPCR (Fig. 2C–E) and semi-quantitative PCR (Figs 2F and S2B) for the expression of HOTAIR, IL-6 and iNOS. Interestingly, treatment with IKK*β* inhibitor down-regulated LPS-induced expression of NF-κB target genes IL-6 (5.4 folds) and iNOS (15.3 folds) along with a decrease in HOTAIR (2.4 folds) expression (Fig. 2C–F). The treatment of SC-154 alone in the absence of LPS has no significant impact on IL-6, iNOS and HOTAIR expression (Fig. 2C–F). These observations suggest that similar to IL-6 and iNOS, HOTAIR expression is also regulated by NF-κB upon LPS-stimulation.

### HOTAIR knockdown abolishes LPS-induced IL-6 and iNOS expression

**Figure 3:**
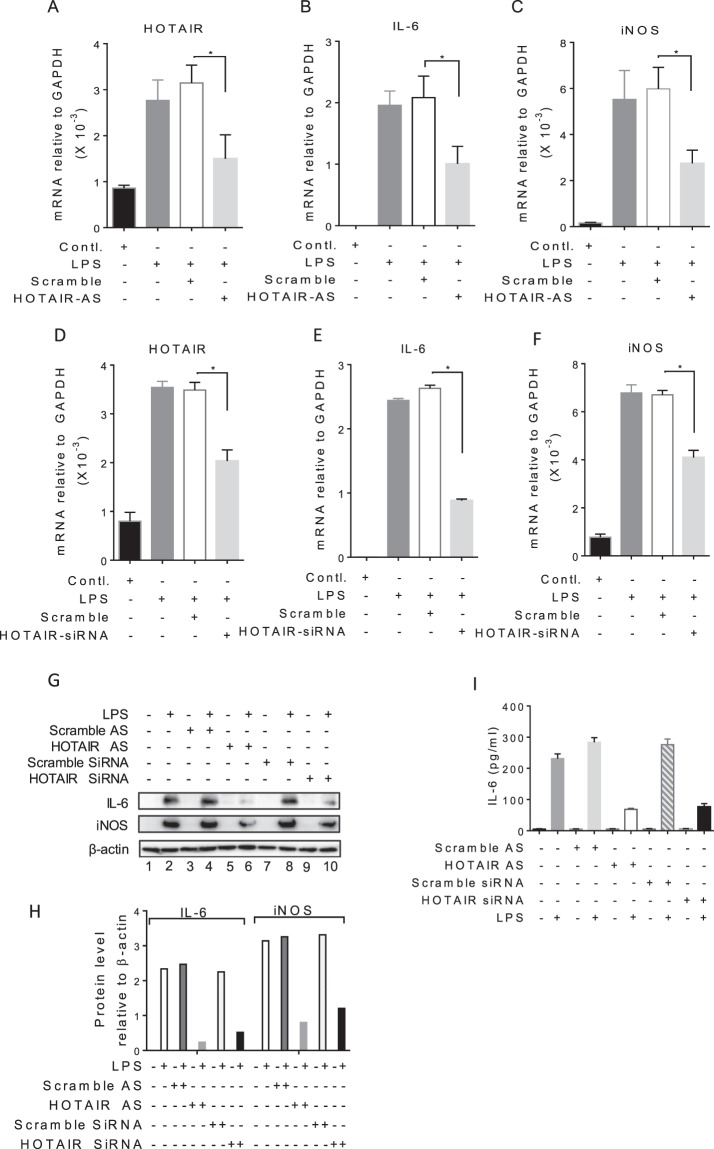
Knockdown of HOTAIR reduces LPS-induced IL-6 and iNOS expressions in macrophages. (**A**–**C**) Antisense-mediated knockdown of HOTAIR followed by treatment with LPS. RAW264.7 cells were transfected with HOTAIR-AS and scramble-AS, then treated with LPS for 4 h. RNA was analyzed by RT-qPCR for the expression of HOTAIR, IL-6, and iNOS. (**D**–**F**) siRNA-mediated knockdown of HOTAIR followed by treatment with LPS. RAW264.7 cells were transfected with HOTAIR-siRNA and scramble, RNA was analyzed RT-qPCR for the expression of HOTAIR, IL-6, and iNOS. GAPDH was used as control. Data represent mean ± SD (n = 3); *p < 0.05, **p < 0.001, ***p < 0.0001. (**G**,**H**) Proteins from HOTAIR-antisense or HOTAIR-siRNA treatment followed by 6 h LPS-treated RAW264.7 cells were analyzed by Western blotting using antibodies against IL-6, iNOS and β-actin (loading control) (**G**). The changes in amounts of IκBα and NF-κB have been quantified by ImageLab5.2.1 software (**H**). (**I**) ELISA for IL-6 expression. HOTAIR was silenced in RAW264.7 macrophages by using HOTAIR-antisense and HOTAIR-siRNA separately, treated with LPS (12 h). Culture media were collected and amount of IL-6 secreted in culture media were measured using ELISA (n = 4).

To investigate any potential contribution of HOTAIR in cytokine regulation and inflammatory response, we knocked down HOTAIR in macrophages and analyzed its impacts on LPS-induced expression of IL-6 and iNOS. In brief, RAW264.7 cells were transfected with HOTAIR-antisense (HOTAIR-AS) and scramble-antisense (control) for 48 h, then treated with LPS (1 µg/mL, 4 h) and RNA was analyzed by RT-qPCR. As expected, LPS treatment induced the expression of HOTAIR as well as IL-6 and iNOS (Fig. 3A–C). The application of HOTAIR-AS knocked down the levels of LPS-induced HOTAIR (Fig. 3A). Scramble-AS did not downregulate HOTAIR level (Fig. 3A). Interestingly, the knockdown of HOTAIR resulted in down-regulation of the LPS-induced expression levels of IL-6 and iNOS significantly (Fig. 3B,C). To further confirm the antisense-specificity of HOTAIR, we knocked down HOTAIR using HOTAIR-specific siRNA (a pool of 4 different siRNAs, targeting a different region 450–800 bp, 800–1050 bp, 1050–1550 bp and 1550–1900 bp of HOTAIR)^67^ and then analyzed it impacts in LPS-induced expression of IL-6 and iNOS. Interestingly, HOTAIR-siRNA knocked down HOTAIR and also down-regulated LPS-induced IL-6 and iNOS expression levels (Fig. 3D–F). To understand further the roles of HOTAIR in immune and inflammatory signaling, we analyzed the expression of additional cytokine and inflammatory genes such as TNFα, MIP-1β and others, in the absence and presence of LPS and under HOTAIR knockdown (antisense or siRNA-treatments) conditions. Interestingly, HOTAIR-knockdown (using antisense or siRNA) also down-regulated the LPS-induced expression of TNFα, MIP-1β and others, indicating further the importance of HOTAIR in immune and inflammatory response (Fig. S3A,B).

In addition to mRNA levels, we also analyzed the protein levels of IL-6 and iNOS in the absence and presence of LPS and under HOTAIR-conditions, using Western blots. As expected, IL-6 and iNOS were increased upon treatment with LPS (compare lanes 1 and 2) and their levels were decreased upon treatment HOTAIR-AS (compare lane 2 and 6) or HOTAIR-siRNA (compare lanes 2 and 10) (Fig. 3G, quantification in H and Supplementary Fig. S3C). Scramble antisense or siRNA has no significant impacts on the LPS-induced expression of IL-6 and iNOS. Furthermore, we also measured the secretion of IL-6 in culture media using ELISA and found that secreted IL-6 level was increased upon treatment with LPS and that were decreased upon treatment HOTAIR-AS or HOTAIR-siRNA (Fig. 3I). Taken together, our analysis demonstrated HOTAIR is required for LPS-induced expression of cytokines and inflammatory response genes in macrophages.

### HOTAIR knockdown results in inactivation of NF-κB

**Figure 4:**
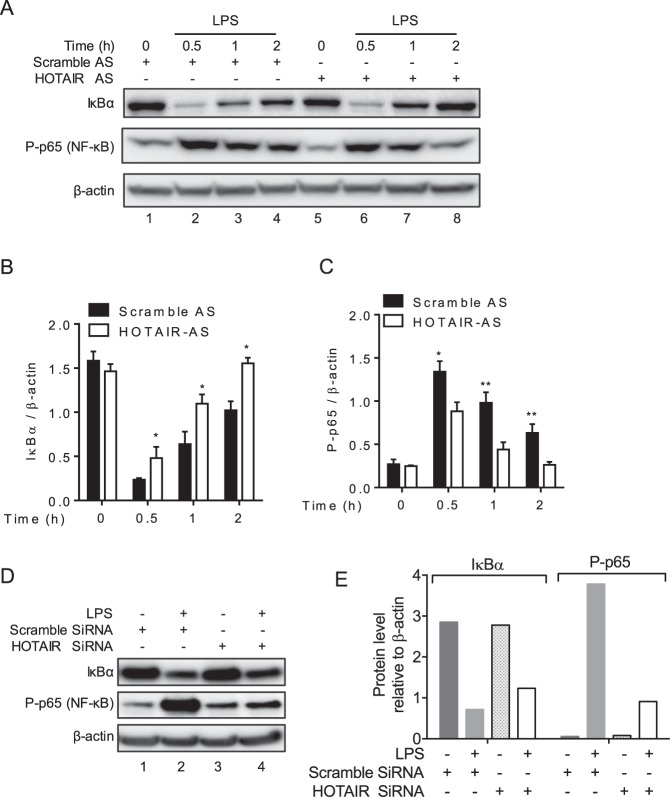
HOTAIR promotes IκBα degradation thereby activates NF-κB. (**A**–**C**) Antisense-mediated knockdown of HOTAIR followed by LPS-treatment. RAW264.7 macrophages were transfected with HOTAIR- or scramble antisense for 48 h, treated with LPS for different time periods (0.5 h, 1 h and 2 h) and protein was isolated. The protein was resolved on SDS-PAGE and immunoblotted with antibody against IκBα, phospho p65 (NF-κB subunit) and β-actin (loading control). The changes in amounts of IκBα and NF-κB have been quantified by ImageLab5.2.1 software (**B**,**C**). (**D**,**E**) siRNA-mediated knockdown of HOTAIR followed by LPS-treatment. Proteins from HOTAIR- or scramble siRNA (48 h-treatment) followed by LPS (1 h) -treated RAW264.7 were analyzed by Western blot using antibody against IκBα, phospho p65 and β-actin. Quantifications (ImageLab5.2.1software) are shown in panel E. Data represent mean ± SD; *p < 0.05, **p < 0.001.

As HOTAIR is required for LPS-induced expression of IL-6 and iNOS, we investigated its potential mechanism of action in this process. Notably, IL-6 and iNOS are regulated via activation of transcription factor NF-κB^82–84^ and therefore, we explored if HOTAIR may be involved in NF-κB activation. We knocked down HOTAIR (using HOTAIR-antisense) in RAW264.7 macrophages and treated with LPS (varying time) and then analyzed the protein levels of IκBα and NF-κB (phospho-p65 subunit) (Figs 4A–C and S4A). Beta-actin was used a loading control. As expected, IκBα level was significantly decreased upon treatment with LPS (compare lane 1 with lanes 2–4, Fig. 4A, quantification of IκBα levels relative to β-actin in Fig. 4B). The degradation of IκBα was highest at 0.5 h of LPS-treatment and it was slightly increased over time (Fig. 4A,B). As expected the level of phospho-p65 (NF-κB subunit) was also increased (NF-κB activation) upon LPS-treatment in comparison to the control (compare lane 1 with lanes 2–4, Fig. 4A and quantification in 4C). The decrease in IκBα and concomitant increase in phospho-p65 upon LPS treatment suggest that LPS-stimulation has induced proteasomal degradation of IκBα resulting in activation of NF-κB. Interestingly, upon treatment with HOTAIR-AS (HOTAIR-knockdown), the level of LPS-induced IκBα degradation was decreased in comparison to LPS alone (compare lanes 2 with 6, 3 with 7, 4 with 8, Fig. 4A,B). HOTAIR-knockdown also reduced phospho-p65 levels compared LPS-alone (compare lanes 2 and 6; 3 and 7; 4 and 8; Fig. 4A,C). To cross verify the impact of HOTAIR-knockdown on deactivation of NF-κB, we also examined the IκBα and NF-κB (phospho-p65 subunit) levels under HOTAIR-siRNA treatment followed by LPS-stimulation (Figs 4D,E and S4B). Interestingly, siRNA-mediated knockdown of HOTAIR resulted in decrease in LPS-induced IκBα degradation and reduced phospho-p65 levels (compare lanes 2 with 4 in Fig. 4D, quantifications in 4E). Scramble-siRNA has no significant impacts on LPS-induced level of IκBα degradation and phospho-p65 (Fig. 4D,E). These observations demonstrate that HOTAIR is required for LPS-induced proteasomal degradation of IκBα and NF-κB activation.

### HOTAIR promotes IκBα degradation and nuclear translocation of NF-κB

**Figure 5:**
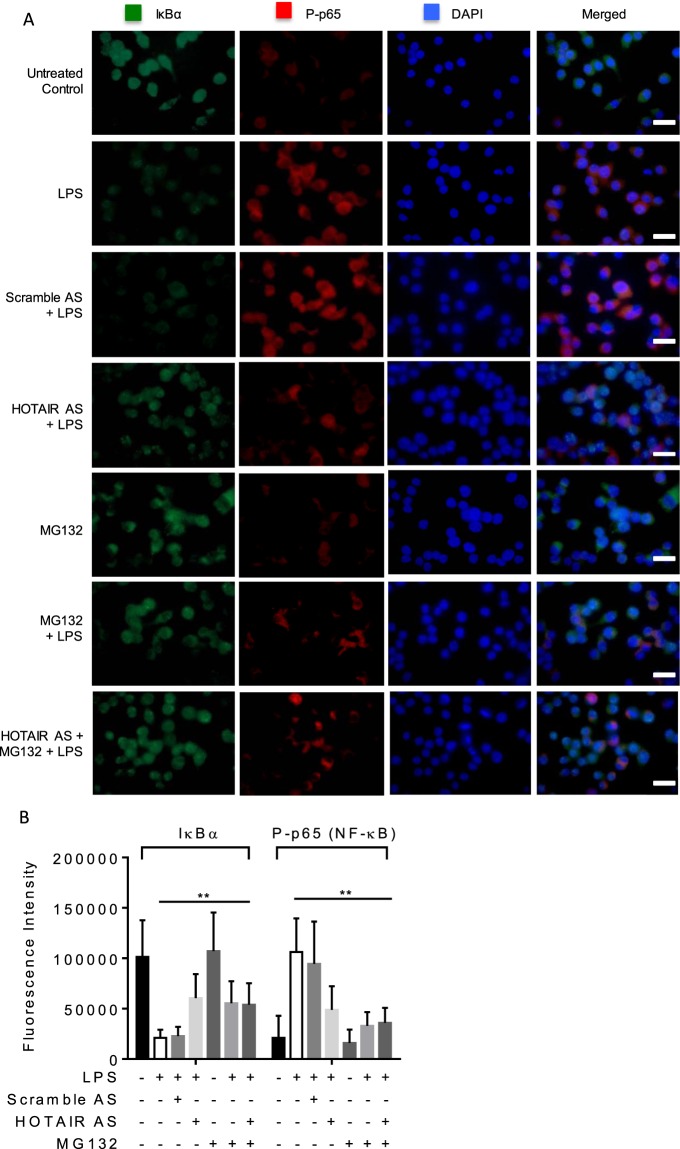
HOTAIR promotes IκBα degradation and nuclear translocation of NF-κB. HOTAIR was silenced in RAW 264.7 cells by using HOTAIR antisense and scramble (control) for 48 h. Additionally, cells were also treated with proteasomal inhibitor MG132 (2 h) alone or in combination with HOTAIR-knockdown and then treated with LPS (1 h). The cells were then fixed with paraformaldehyde and immunostained with antibody against IκBα and phospho NF-κB (P-p65), and counterstained with DAPI. Images were taken by fluorescence microscope (Nikon ECLIPSE TE2000-U) (**A**) and fluorescence intensity showing the expressions of IκBα and phospho-p65 was quantified and plotted by ImageJ software (**B**). Data represent mean ± SD; *p < 0.05, **p < 0.001.

To further understand the function of HOTAIR in NF-κB activation, we measured the co-expression of IκBα and phospho-p65 (NF-κB) in macrophages by using immunofluorescence assay. We knocked down HOTAIR in RAW264.7 cells by using HOTAIR-AS and scramble-AS (control) and then treated LPS. Control and antisense-treated cells were subjected to immunostaining with IκBα and phospho-p65 (NF-κB) antibodies. Cell nucleus was stained with DNA binding dye DAPI. We observed that IκBα protein levels were higher while phospho NF-κB protein levels were lower in unstimulated (in the absence of LPS) control cells (Fig. 5A, top panel, quantification of the immunofluorescence staining images are shown in Fig. 5B). However, when stimulated with LPS we found a decrease in IκBα levels and increase in phospho NF-κB in macrophages compared to LPS-untreated control cells (compare top two panels, Fig. 5A). Interestingly, upon HOTAIR-knockdown (HOTAIR-AS and LPS treatments), IκBα level was increased in comparison to LPS-treatment alone (compare panels 4 and 2, Fig. 5A,B). The level of phospho-p65 protein (NF-κB) was decreased concomitantly upon knockdown of HOTAIR (HOTAIR-AS and LPS treatment) relative to LPS alone (compare panels 4 and 2, Fig. 5A,B). Scramble antisense has no significant impact on LPS-induced expression of IκBα and phospho-p65 levels (Fig. 5A,B). These results further indicate that HOTAIR is required for LPS-induced proteasomal degradation of IκBα and activation of NF-κB in immune cells. Notably, to further understand the potential involvement of HOTAIR in proteasomal degradation of IκBα, we applied a well-known proteasomal inhibitor MG132 followed by treatment with LPS in the presence and absence of HOTAIR knockdowns. Interestingly, application of MG132 (followed by treatment with LPS) also inhibited the level of LPS-induced degradation of IκBα and lowered phospho-p65 level (compare panels 6 and 2, Fig. 5A,B). The effects of independent treatment of MG132 or HOTAIR-AS on inhibition of LPS-induced degradation of IκBα and phospho-p65 level are comparable (compare panels 6 and 4, Fig. 5A,B). The application of MG132, in combination with HOTAIR-AS followed by LPS-treatment, showed no further significant impact in comparison to MG132 or HOTAIR-AS treatments alone (compare panels 7 with 6 and 4, respectively, Fig. 5A,B). Overall, these studies demonstrate that HOTAIR plays potential roles in the regulation of proteasomal degradation of IκBα and subsequent activation of NF-κB in response to LPS in immune cells.

### HOTAIR knockdown abolishes the recruitment of NF-κB at IL-6 and iNOS promoters

**Figure 6:**
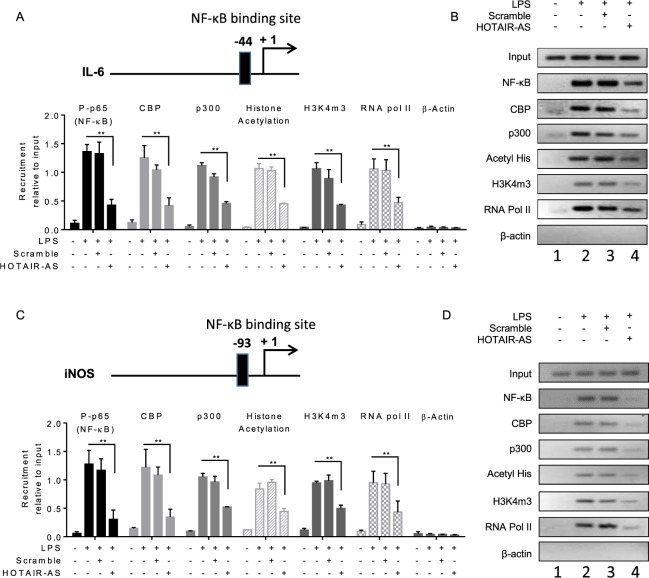
Knockdown of HOTAIR reduces the recruitment of transcription factors and coactivators at NF-κB binding sites on IL-6 and iNOS promoters. RAW264.7 macrophage cells were transfected with HOTAIR or scramble-antisense, then treated with LPS (1.5 h). Cells were then fixed with formaldehyde and subjected to ChIP assay using antibodies specific to phospho-p65, CBP, p300, histone acetylation, H3K4m3, RNA pol II and β-actin (control). The immunoprecipitated DNA fragments were analyzed by qPCR (panels A shows the ChIP analysis for IL-6, and C for iNOS) and semi-quantative PCR (panels B and D) using primers specific to the NF-κB binding regions on IL-6 and iNOS promoters. The location of NF-κB binding sites at the IL-6 and iNOS promoters are shown at the top of panels A and C respectively. Each experiment was repeated at least thrice (n = 3). Data represent mean ± SD; *p < 0.05, **p < 0.001.

The pro-inflammatory cytokine and inflammatory response genes such as IL-6 and iNOS are regulated via transcription factor NF-κB^85,86^. The promoters of IL-6 and iNOS contain NF-κB binding sites (NF-κB response elements) (Figs 6A,C and S5A,B). As HOTAIR is required for NF-κB activation and expression of NF-κB regulated genes IL-6 and iNOS expression, we analyzed HOTAIR-dependent recruitment of NF-κB at the NF-κB binding sites present in IL-6 and iNOS promoters as a function of LPS-treatment, using chromatin immunoprecipitation (ChIP) assay. Briefly, control and HOTAIR-knocked down RAW264.7 treated with LPS were subjected ChIP using phosphorylated p65 (NF-κB subunit) and β-actin (control) and ChIP DNA fragments were PCR-amplified using primers specific to NF-κB binding sites present in IL-6 and iNOS promoters^60,87^. Interestingly, these analyses demonstrated that phospho-p65 (NF-κB) levels were enriched at the IL-6 and iNOS promoters (NF-κB response element regions) upon treatment with LPS and these LPS-induced NF-κB binding was reduced upon HOTAIR-knockdown in both IL-6 and iNOS promoters (see p-65 ChIP qPCR data in Fig. 6A,C and also compare lanes 2 and 4, Fig. 6B,D for regular PCR analysis). Notably, along with NF-κB, there are other coactivators which are associated with LPS-induced IL-6 and iNOS expression. For example, histone acetyltransferase CBP/p300 is known to interact with NF-κB to regulate NF-κB target genes^88–90^. Our ChIP analysis demonstrates that similar to NF-κB, the levels of CBP/p300 were also enriched at IL-6 and iNOS promoters and these LPS-induced enrichments were alleviated upon knockdown of HOTAIR (Fig. 6A,B for qPCR analysis and C-D for regular PCR analysis, compare lanes 2 and 4). Scramble antisense treatment has no significant impact on the LPS-induced enrichment of NF-κB and CBP/p300 at the IL-6 and iNOS promoters. As a control, we measured β-actin (antibody control), but no enrichment was observed irrespective of the LPS or HOTAIR-AS treatment (Figs 6A–D, S5A,B). These observations suggest that HOTAIR plays key roles in LPS-induced NF-κB activation and hence its enrichment at the promoters of NF-κB target genes such as IL-6 and iNOS to regulate their expression.

Histone H3K4-trimethylation and histone acetylation are also well known marks for gene activation^60,87,91–93^. ChIP analysis demonstrated that levels of H3K4-trimethylation and histone acetylation as well as the level of RNA polymerase II (RNA pol II) were enriched at IL-6 and iNOS promoters in the presence of LPS and this was decreased upon HOTAIR knockdown (Fig. 6A–D). These observations suggested that HOTAIR is required for promoter activation (H3K4-methylation and histone acetylation) of IL-6 and iNOS and this is mediated via activation of NF-κB followed by recruitment of NF-κB and its coregulators including histone methyl-transferases and histone acetyl-transferases at the target gene promoters. Taken together our ChIP analysis demonstrated that LPS-induced expression of IL-6 and iNOS are regulated via transcription factors NF-κB, CBP/p300 and other coactivators and this is regulated by HOTAIR via regulation of NF-κB activation.

### HOTAIR expression is induced by LPS in primary macrophages and is required for LPS-induced cytokine expression

**Figure 7:**
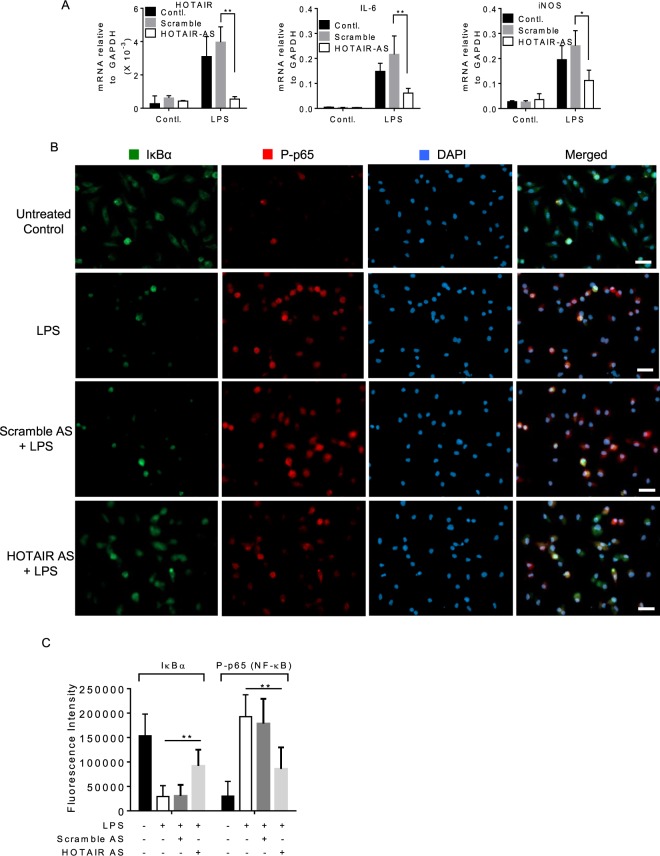
HOTAIR expression is induced by LPS in primary macrophages (Bone marrow derived macrophages, BMDM). (**A**) BMDM cells were treated by HOTAIR-AS and scramble-AS followed by LPS treatment and RNA was extracted. The expression of HOTAIR, IL-6, and iNOS was measured by real time PCR. (**B**,**C**) BMDM cells were treated by HOTAIR-AS and scramble-AS, treated with LPS and were immunostained with antibodies against IκBα and phospho NF-κB (P-p65), and counterstained with DAPI to visualize the nucleus. Images were taken by fluorescence microscope (Nikon ECLIPSE TE2000-U) (**B**) and fluorescence intensity showing the expressions of IκBα and phospho NF-κB (P-p65) was quantified by ImageJ software and plotted in panel C. Each experiment was repeated at least thrice (n = 3). Data represent mean ± SD; *p < 0.05, **p < 0.001.

We investigated further the importance of HOTAIR in cytokine expression and immune response in primary macrophages, bone marrow derived macrophages. Briefly, BMDM cells were treated with LPS (1 µg/mL, for 4 h) in the presence and absence of HOTAIR and scramble (control) antisense, RNA was analyzed by RT-qPCR. Interestingly, the levels of IL-6 and iNOS as well as HOTAIR expression were induced upon treatment with LPS in BMDM (compare 1^st^ and 4^th^ bar graphs in each panel, Fig. 7A). Application of HOTAIR-AS resulted in significant knockdown of LPS-induced HOTAIR expression level (compare 4^th^ or 5^th^ with 6^th^ bar graphs, left panel, Fig. 7A). Importantly, the levels of LPS- induced expression of IL-6 and iNOS were decreased upon HOTAIR knockdown (compare 4^th^ or 5^th^ with 6^th^ bar graphs for IL-6 and iNOS panels, Fig. 7A). HOTAIR-AS has no significant impact on IL-6 and iNOS expression in the absence of LPS and also scramble antisense has no significant impacts on LPS-induced IL-6 and iNOS expression (Fig. 7A). These observations demonstrated that HOTAIR expression is induced upon LPS-stimulation in primary macrophages and it is required for the LPS-induced expression of IL-6 and iNOS.

HOTAIR-knocked down and LPS-treated BMDM cells were also analyzed by immunostaining to understand the function of HOTAIR in NF-κB activation using similar experiments outlined in Fig. 5. Interestingly, we found that IκBα protein levels were higher in the control BMDM (no LPS) and this was decreased upon treatment with LPS (Fig. 7B). The level of NF-κB (phospo-p65) protein was increased upon treatment with LPS (Fig. 7B). Interestingly, we also found that upon HOTAIR-knockdown, the level of LPS-induced decrease in IκBα protein level was rescued significantly while LPS-induced phospho-p65 NF-κB protein level was decreased. Scramble antisense has no significant impacts on LPS-induced expression of IκBα and NF-κB (phospho-p65) protein levels. Quantification of immunofluorescence images showing the expression of IκBα and NF-κB (phospho-p65) protein levels under different treatments are shown in Fig. 7C. These observations based on primary macrophage analysis further demonstrate that HOTAIR plays a critical role in LPS-induced NF-κB activation and immune response.

## Discussion

The human genome contains about 3 billion base pairs of which only 1.5% codes for proteins^94–96^. The ENCODE project has suggested that more than 80% of the genome is functionally active and codes for ncRNA and regulatory sequences^96,97^. NcRNAs, even though are not translated into proteins, appear to play critical roles in a variety of cellular and physiological processes including gene regulation, cells signaling, differentiation and development^37,98^. NcRNAs are misregulated in human diseases^37,99^. Increasing numbers of studies indicate that lncRNAs are associated with immune signaling and inflammatory response^100^. LncRNAs are expressed in immune cells including monocytes, macrophages, dendritic cells, neutrophils, T cells and B cells^101^. For example, lncRNA lincR-Ccr2–5′AS is associated with CD4^+^ T cell differentiation^98^. LncRNAs are linked to pathogen-response pathways such as lincRNA-Cox2 expression is elevated upon activation of the toll-like receptors in bone-marrow-derived dendritic cells and macrophages^31,39,98^. LincRNA-Cox2 is required for the induction of other immune-related genes, such as IL-6, Tlr1, and IL-23a in bone marrow-derived macrophages by Pam3CSK4 treatment^39,102^. NRON (non-protein coding RNA, repressor of NFAT) acts as a transcription regulator for immune regulation by inhibiting nucleocytoplasmic shuttling of NFAT (Nuclear Factor of Activated T cells)^103^. Lethe acts as a decoy lncRNA and is a negative feedback inhibitor of NF-kB signaling in inflammation by being increased with proinflammatory cytokines such as TNFα and IL-1β^31,104^. THRIL (TNFα and heterogeneous nuclear ribonucleoprotein L related immunoregulatory lincRNA) plays immunoregulatory roles by binding with heterogeneous nuclear ribonucleoprotein L (hnRNPL) to induce the expression of TNFα, IL-6, IL-8, CXCL10, CCL1 and CSF1^39,105,106^. Here, we investigated the importance of lncRNA HOTAIR in immune and inflammatory response in macrophages.

Our studies demonstrate that HOTAIR expression is induced in macrophage cells in response to LPS treatment. LPS, present on the Gram-negative bacteria cell wall, is one of the most potent pathogen-associated molecular patterns (PAMPs) known and responsible for the inflammatory response observed during endotoxic shock^83,107–109^. LPS stimulation induces variety of cytokines, chemokines and inflammatory genes such as ILs, TNFs, interferons (IFNs), iNOS etc^3,85,110–113^. In our studies, we observed that LPS-treatment (macrophage RAW264.7) induced the expression of various cytokines and inflammatory genes including IL-6, iNOS, TNFα, MIP-1B and others along with HOTAIR. Interestingly, we also observed that HOTAIR is required for the LPS-induced cytokines and inflammatory genes expression. Antisense or siRNA-mediated knockdown of HOTAIR abolished the LPS-induced activation of IL-6, iNOS, TNFα, and MIP-1B. HOTAIR-knockdown down regulated the expression of IL-6 and iNOS, both at mRNA and protein levels. Independent knockdowns of HOTAIR with antisense or siRNAs targeting different regions of HOTAIR confirmed the HOTAIR-target specificity. These observations demonstrate that HOTAIR is a critical player in cytokine expression and inflammatory response in macrophages upon LPS-stimulation.

It is well known that LPS-induces activation of TLRs which activates down-stream signaling cascades and this ultimately results in degradation of IκBα and activation of NF-κB; the NF-κB activation triggers its target gene expression and induces immune and inflammatory response^114–118^. To investigate if LPS-induced HOTAIR expression is regulated via NF-κB activation and if HOTAIR is required for LPS-induced NF-κB activation and its target gene expression, we blocked NF-κB activation via application of IKKβ-inhibitor (SC-514). We observed that upon treatment with IKKβ-inhibitor, the levels of phospho-IκBα as well as phospho-p65 were decreased. LPS-induced HOTAIR expression is also reduced upon IKKβ-inhibition. Notably, the kinase IKKβ phosphorylates IκBα at Ser 32/36, which triggers its polyubiquitination and proteasomal degradation, resulting in release and activation of NF-κB^6,77^. Activation of NF-κB is accompanied by phosphorylation of its subunit p65 (at Ser 536)^80,81^. Thus, the decrease in phospho-IκBα as well as phospho-p65 level indicated effective inhibition of NF-κB by SC-514. The decrease in HOTAIR expression upon IKKβ-inhibition indicated potential roles of NF-κB in LPS-induced HOTAIR expression. Furthermore, our biochemical and immunofluorescence studies demonstrated that HOTAIR is required for LPS-induced degradation of IκBα and activation of NF-κB and its nuclear translocation. HOTAIR-knockdown inhibited the LPS-induced degradation of IκBα and decrease on phosphorylation of p-65 (NF-κB), similar to the effects observed upon independent application of a proteasomal inhibitor MG132. This observation indicated that HOTAIR may be important for facilitating LPS-induced degradation of IκBα and activation of NF-κB. ChIP analysis demonstrated that HOTAIR is required for the recruitment of NF-κB and its coregulators at NF-κB target genes promoters regulating their expression. Experiments using primary macrophages isolated mouse bones further support that HOTAIR is induced by LPS and its expression is required for LPS-mediated NF-κB activation and hence IL-6 and iNOS expression. Thus, based on our biochemical studies in RAW264.7 macrophage cell lines and primary cells (BMDM) we demonstrated that HOTAIR plays a central role in NF-κB activation and pro-inflammatory response in immune cells upon stimulation with LPS.

**Figure 8:**
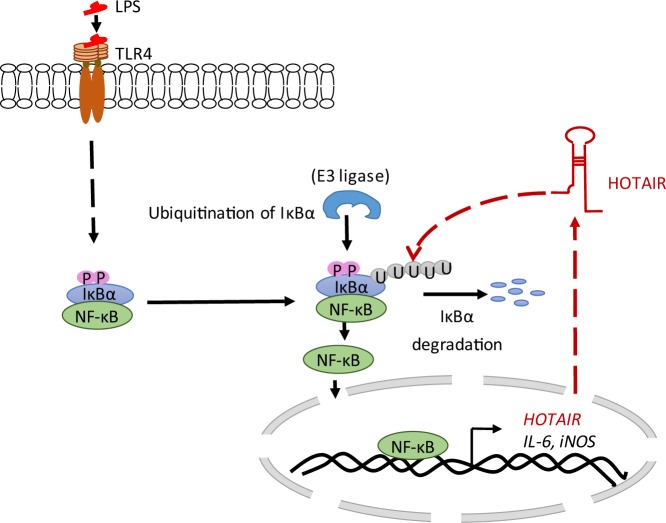
Proposed model for LPS-mediated HOTAIR induction in immune cells. When TLR4 senses LPS, NF-κB is activated that induces IL-6, iNOS and HOTAIR expression. In turn, HOTAIR facilitates IκBα degradation and enhances NF-κB activation, nuclear translocation and binding to NF-κB regulated genes (pro-inflammatory genes such as IL-6, iNOS) inducing their expressions.

Notably, lncRNA HOTAIR is well known for its functions as repressor via interaction with chromatin modifying enzymes such as histone methylase EZH2 containing complex PRC2 and H3K4-demethylase LSD1^45,119–121^. HOTAIR facilitates the recruitment of PRC2 and LSD1 at the target genes and induces gene silencing^37,122,123^. HOTAIR is also implicated in DNA damage response^124^, proteasomal degradation via assembling E3-ubiquitin ligases as associated neuronal siganling^53,125^ HOTAIR induces ubiquitin-mediated proteolysis via interaction with E3 ubiquitin ligases Dzip3 and Mex3b, along with their respective ubiquitination substrates Ataxin-1 and Snurportin-1^53^. This leads to the degradation of Ataxin-1 and Snurportin-1^37,53,126^. A recent study also demonstrated that HOTAIR is involved in NF-κB activation and DNA damage response in ovarian cancer cells^124^. HOTAIR is overexpressed in variety of cancers^127^. Previous studies from our lab show that HOTAIR is required for the viability of breast cancer cells and its expression is transcriptionally regulated by estradiol via coordination of estrogen receptors (ERs) and ER-coregulators such as MLL (mixed lineage leukemia)-family of histone methyltransferases and CBP/p300 in breast cancer cells^55,56,60,128^. Here, we have demonstrated that HOTAIR expression is induced in immune cells and is required for pathogen-induced activation of cytokine expression and pro-inflammatory response. HOTAIR plays critical roles in IκBα degradation, which results in NF-κB activation followed by its target gene (cytokine, chemokines, and pro-inflammatory genes) expression. A model, showing the potential roles of HOTAIR in regulation of IκBα degradation and hence NF-κB activation, its translocation into the nucleus and binding to the target gene promoters inducing their expressions, is shown in Fig. 8. The detailed mechanism by which HOTAIR regulates IκBα degradation remains elusive. Overall, our observations demonstrate for the first time that HOTAIR plays a central role in inflammation and immune signaling in immune cells and this may shed light into the novel immune signaling pathway that may aid development of novel therapeutics.

## Electronic supplementary material

Supplementary figure and legends
