# Supplementary material for: LncRNA HOTAIR regulates lipopolysaccharide-induced cytokine expression and inflammatory response in macrophages
Source: Sci Rep. 2018 Oct 23;8:15670. doi: 10.1038/s41598-018-33722-2 (PMC6199307; doi:10.1038/s41598-018-33722-2)
Supplement: Supplementary file 1 — Supplementary figure and legends [file 41598_2018_33722_MOESM1_ESM.pdf]

Supplementary informations

LncRNA HOTAIR regulates lipopolysaccharide-induced cytokine expression and inflammatory response in macrophages

Monira Obaid[1], S. M. Nashir Udden[2], Paromita Deb[1], Nadine Shihabeddin[1], Md Hasan Zaki[2], and Subhrangsu S. Mandal[1]*

[1]Department of Chemistry and Biochemistry, The University of Texas at Arlington, Arlington, Texas 76019. [2]Department of Pathology, The University of Texas Southwestern Medical Center, Dallas, Texas 75390.

*Corresponding author. E-mail: smandal@uta.edu; Fax: 817-272-3808

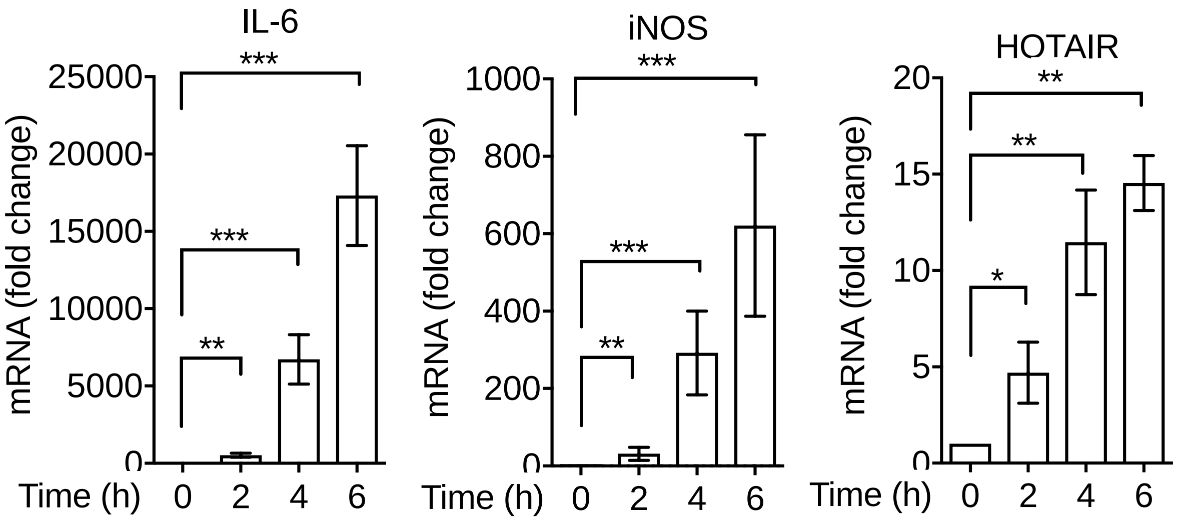

Fig S1A: LPS induces HOTAIR expression in macrophages. RAW264.7 cells were treated with LPS (1 μg/mL) for varying period of time, total RNA was isolated, reverse transcribed to cDNA and analyzed by qPCR for expression of IL-6, iNOS, and HOTAIR. Data represent mean ± SD (n=3); **p < 0.001, ***p < 0.0001.

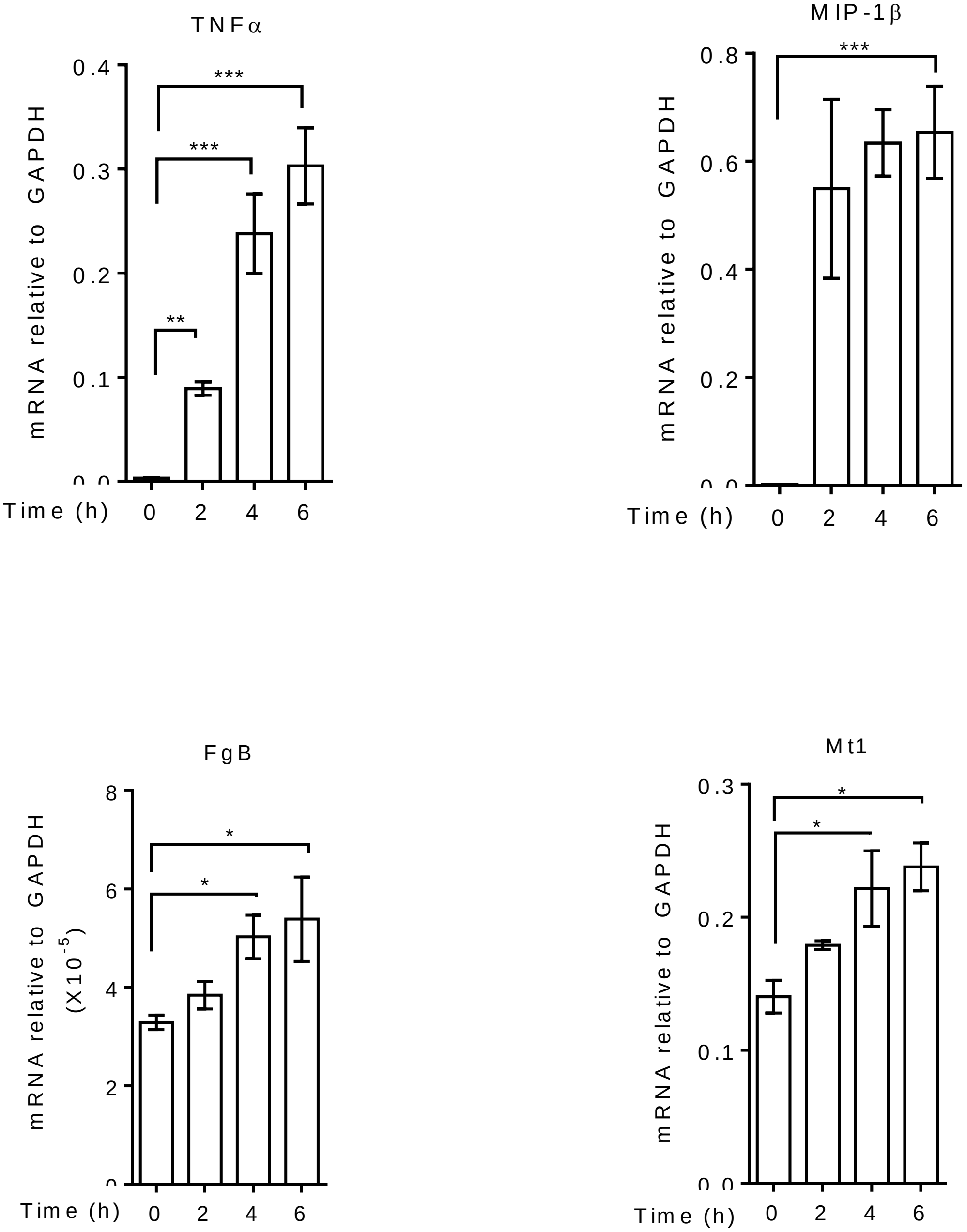

Fig S1B: LPS induces expressions of TNFα, MIP-1B, FgB and Mt1 in macrophages. RAW264.7 cells were treated with LPS (1 µg/mL) for varying period of time, total RNA was isolated, reverse transcribed to cDNA and analyzed by qPCR for expression of TNFα, MIP-1B, FgB and Mt1. Each experiment was repeated at least with three parallel replicates. GAPDH was used as loading control. Data represent mean ± SD (n=3); $*p < 0.05$, $**p < 0.001$, $***p < 0.0001$.

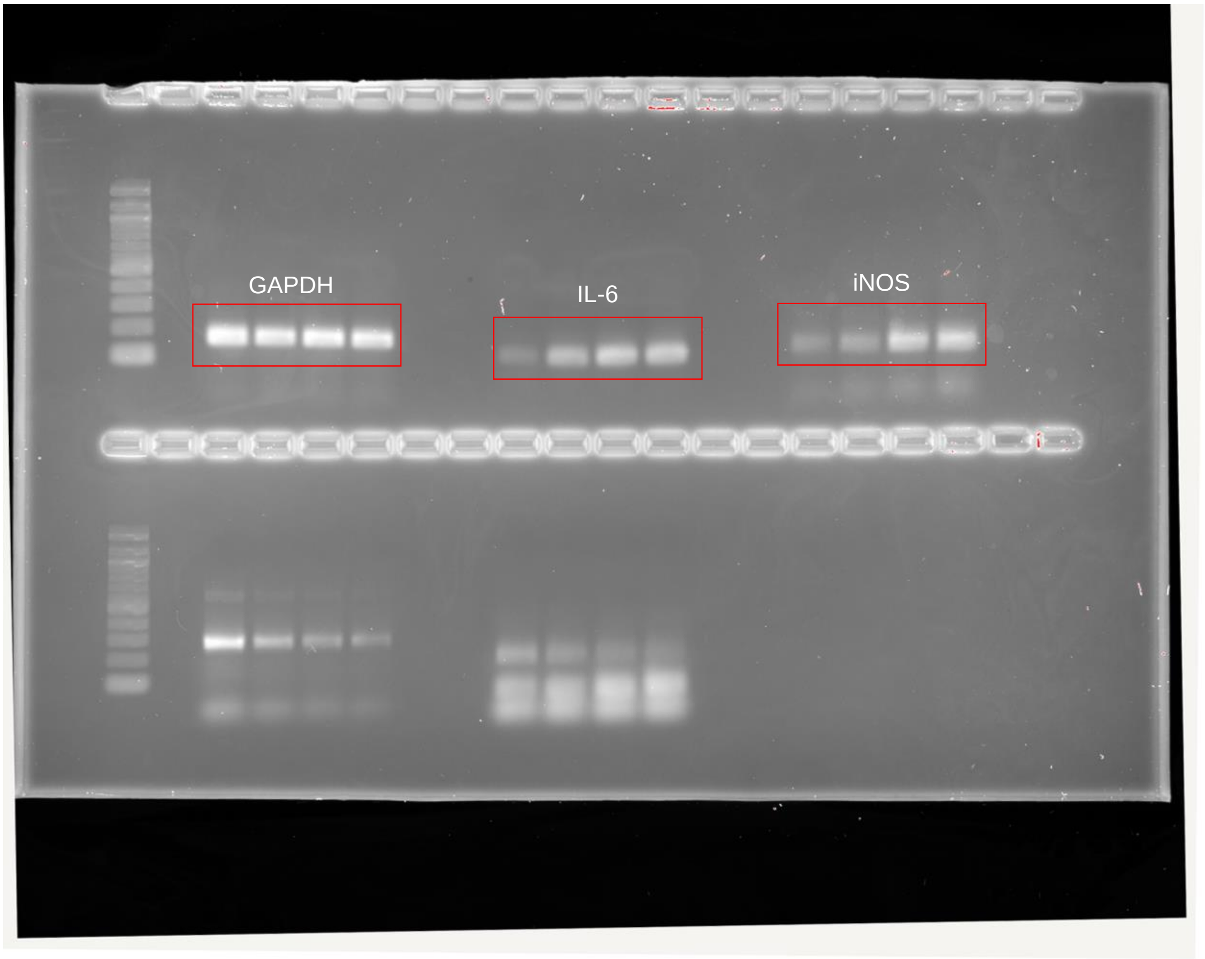

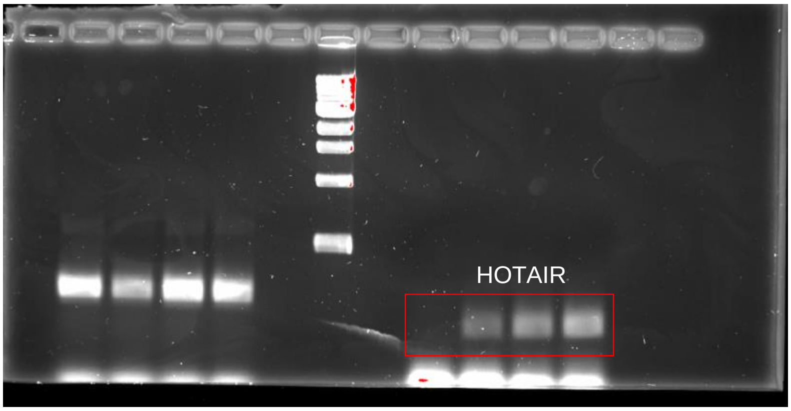

Figure S1C. LPS induces HOTAIR expression in macrophages. RAW264.7 cells were treated with LPS (1 μg/mL) for varying period of time, total RNA was isolated, reverse transcribed to cDNA and analyzed by semi-quantitative PCR and agarose gel. Figure 1D was cropped from the above gels.

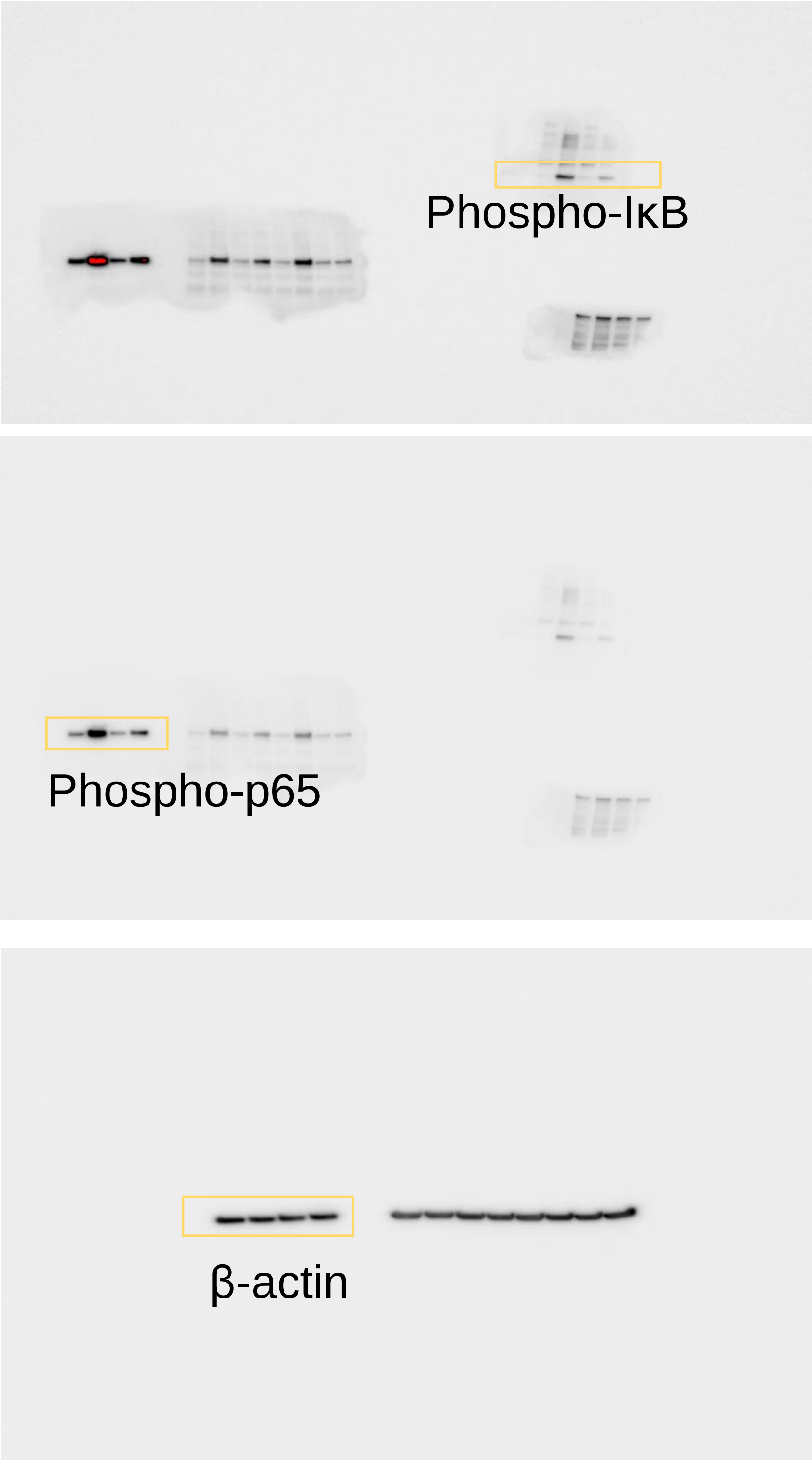

Figure S2A. Inhibition of NF-κB by IKKβ inhibitor (SC-514). RAW264.7 were pre-treated with IKKβ inhibitor (25μM, SC-514, Sigma) for 1h, and after then treated with LPS (1 μg/ml), IKKβ inhibitor individually and combinedly, and kept untreated for 1h. Protein was collected and the protein was resolved on SDS-PAGE and immunoblotted with antibody against the indicated proteins. Figure 2A was cropped from the above images.

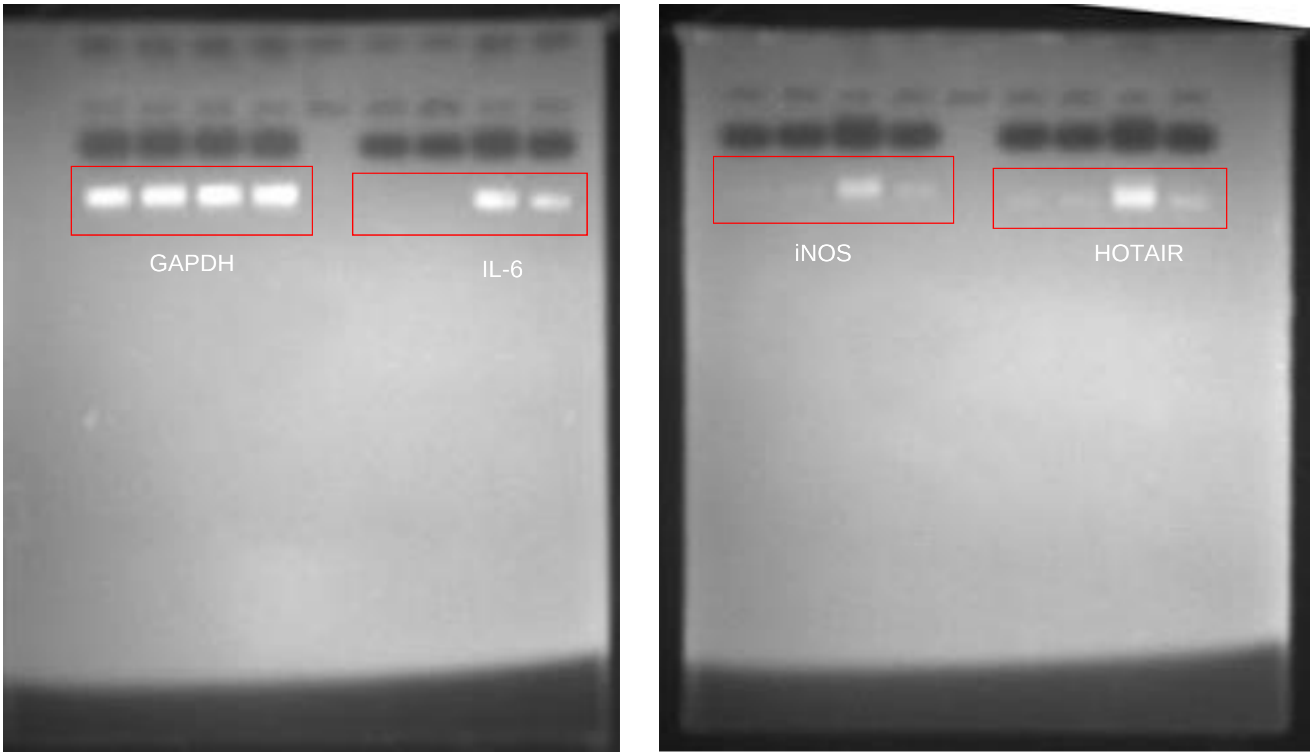

Figure S2B. Inhibition of NF-kB downregulates LPS-induced HOTAIR expression in macrophages. RAW264.7 cells were initially treated with IKKβ-inhibitor SC514 (for 1 h) and then treated with LPS for additional 4 h. RNA was isolated and reverse transcribed to cDNA and analyzed by semi-quantitative PCR and agarose gel. Figure 2F was cropped from the above gels.

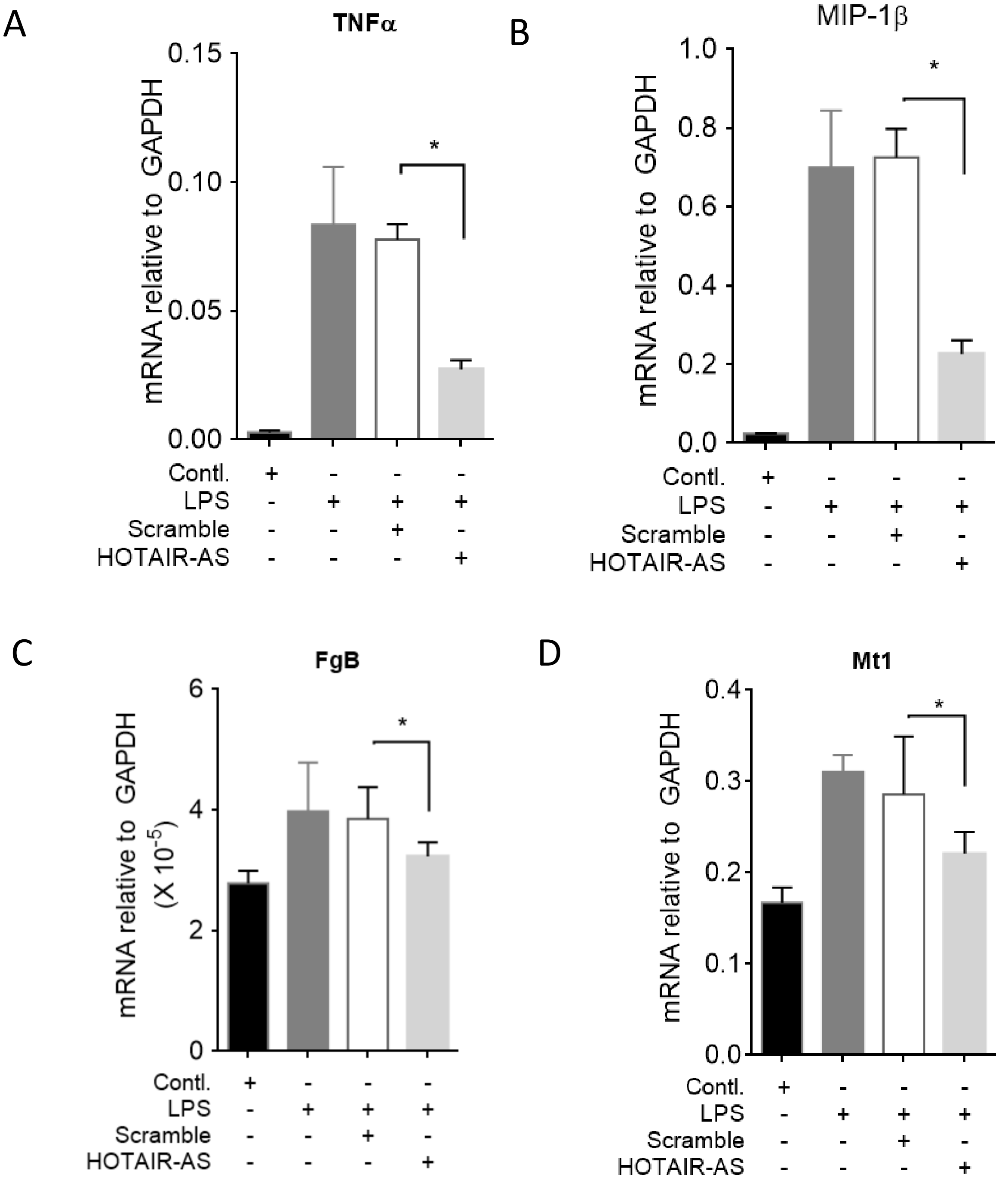

Fig S3A: Antisense mediated Knockdown of HOTAIR reduces LPS-induced TNFα, MIP-1B, FgB and Mt1 expression in macrophages. RAW264.7 macrophage cells were transfected with HOTAIR and scramble-antisense, then treated with LPS for 4 h. RNA was reverse transcribed and expression of TNFα (A), MIP-1B (B), FgB (C) and Mt1 (D) were measured by real time PCR.

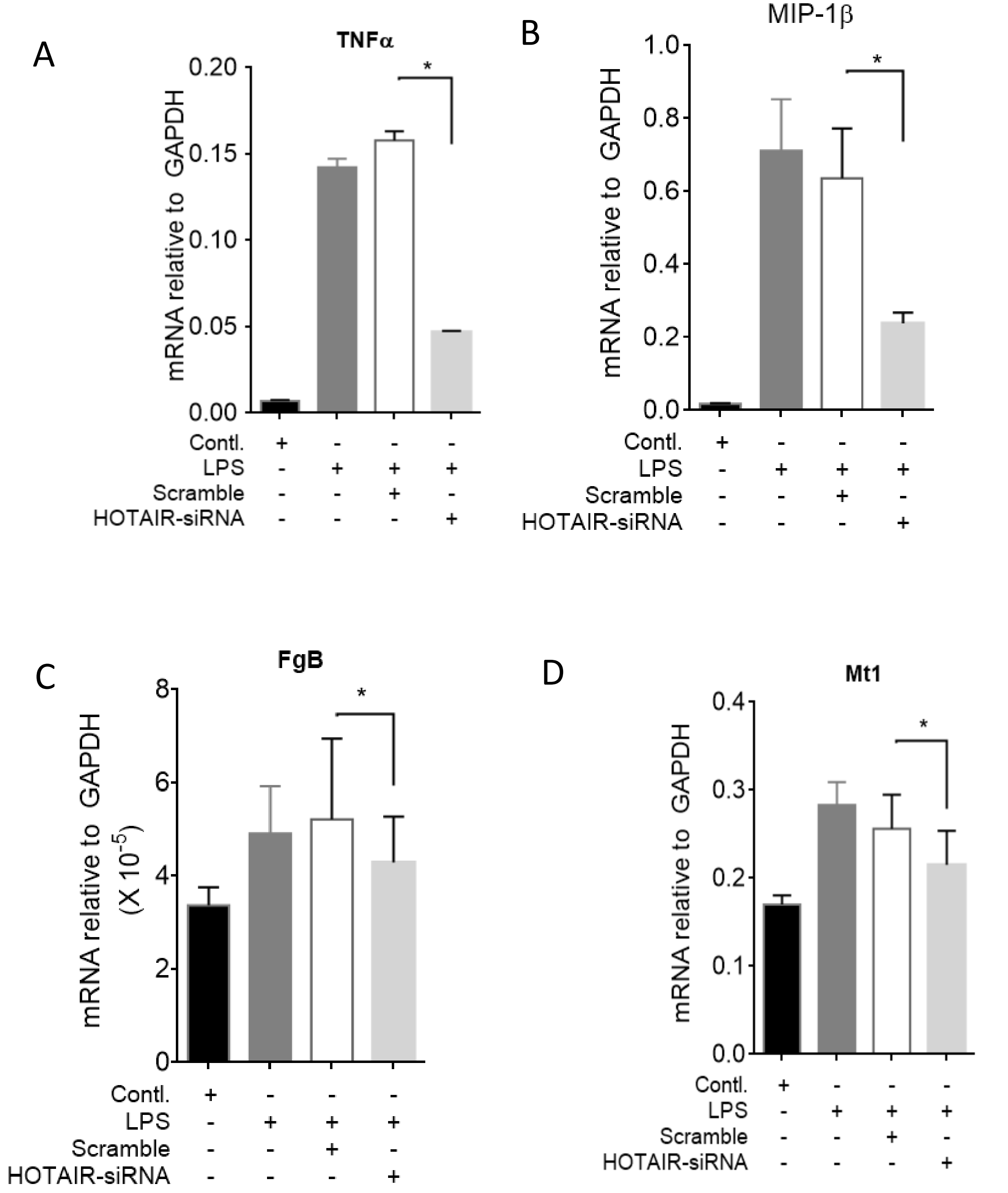

Fig S3B: SiRNA mediated Knockdown of HOTAIR reduces LPS-induced TNFα, MIP-1B, FgB and Mt1 expression in macrophages. RAW264.7 macrophage cells were transfected with HOTAIR and scramble-siRNA, then treated with LPS for 4 h. RNA was reverse transcribed and expression of TNFα (A), MIP-1B (B), FgB (C) and Mt1 (D) were measured by real time PCR.

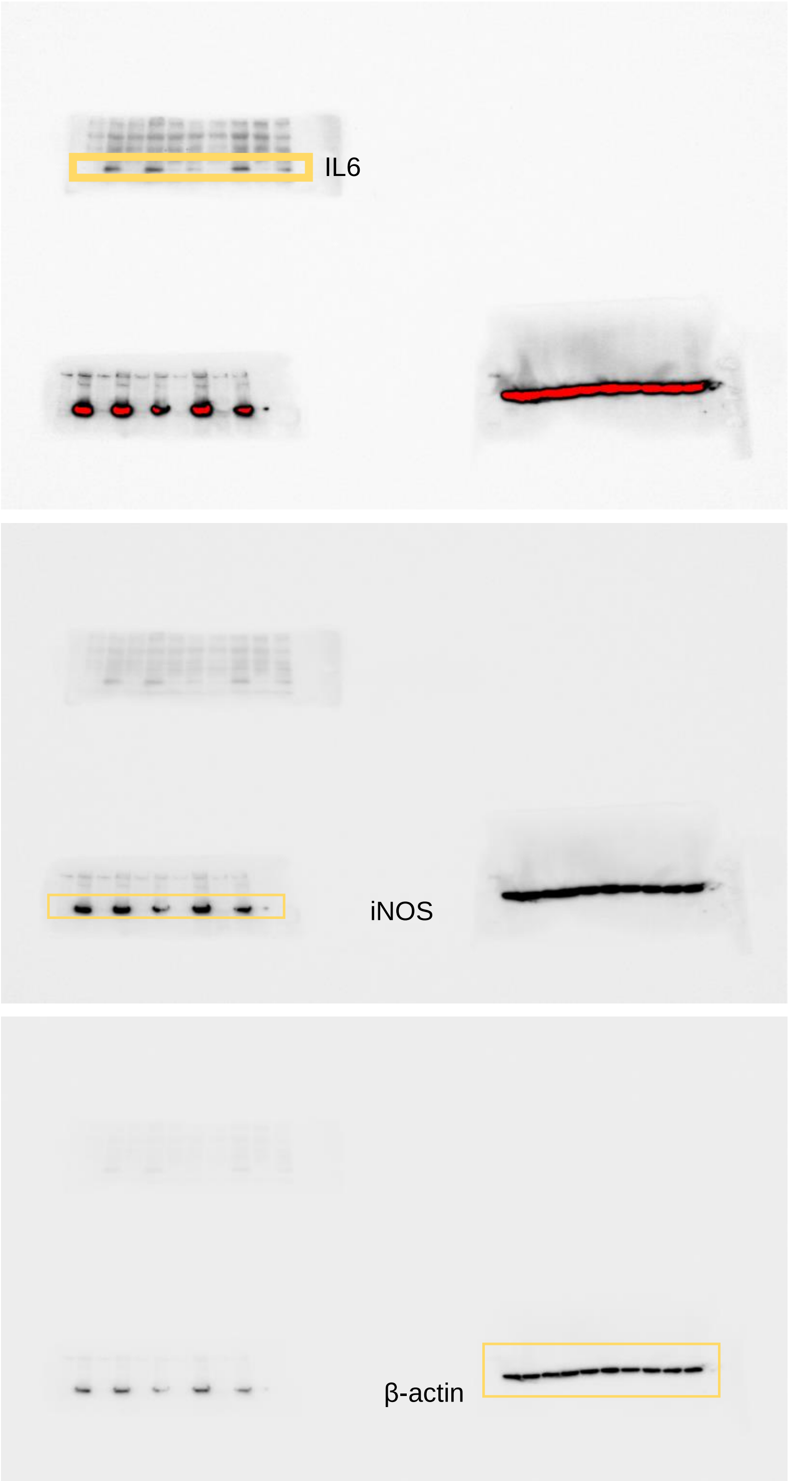

Figure S3C. Knockdown of HOTAIR reduces LPS-induced IL-6 and iNOS expressions in macrophages. HOTAIR was silenced in RAW264.7 macrophages by using antisense (AS) HOTAIR and HOTAIR specific SiRNA 48h following transfection. Cells were stimulated with LPS for 6h or kept untreated, harvested and protein was isolated. The protein was resolved on SDS-PAGE and immunoblotted with antibody against the indicated proteins. Figure 3G was cropped from the above images.

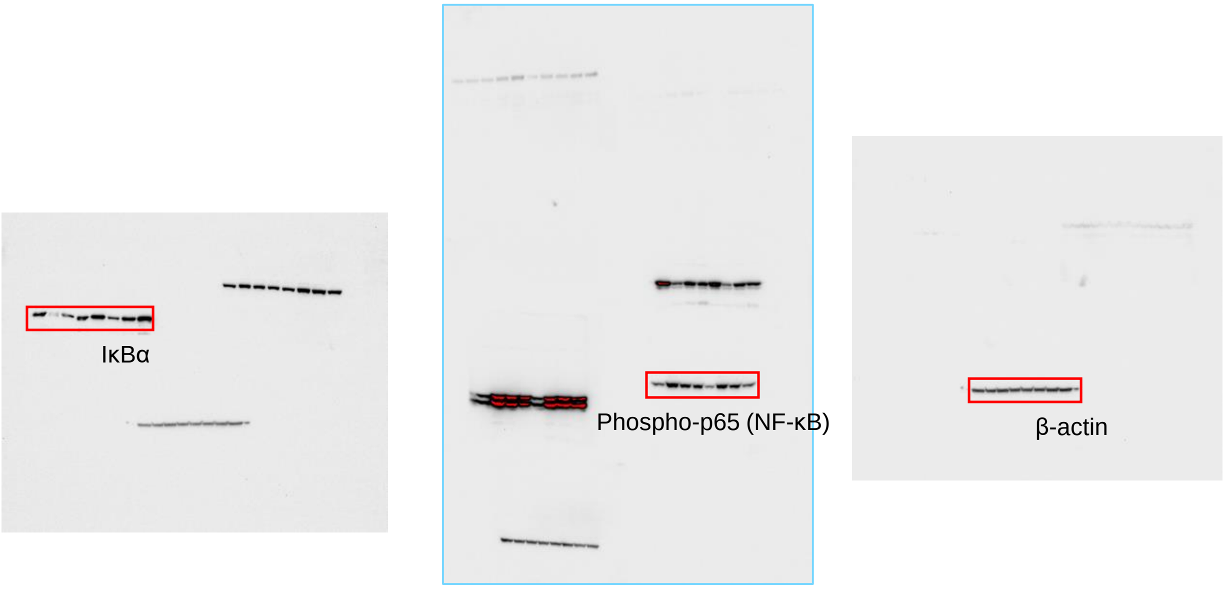

Figure S4A. Antisense mediated knockdown of HOTAIR. RAW264.7 macrophages were transfected with HOTAIR- or scramble antisense for 48 h, treated with LPS for different time periods (0.5 h, 1 h and 2 h) and protein was isolated. The protein was resolved on SDS-PAGE and immunoblotted with antibody against IκBα, phospho p65 (NF-κB subunit) and β-actin (loading control). Figure 4A was cropped from the above images.

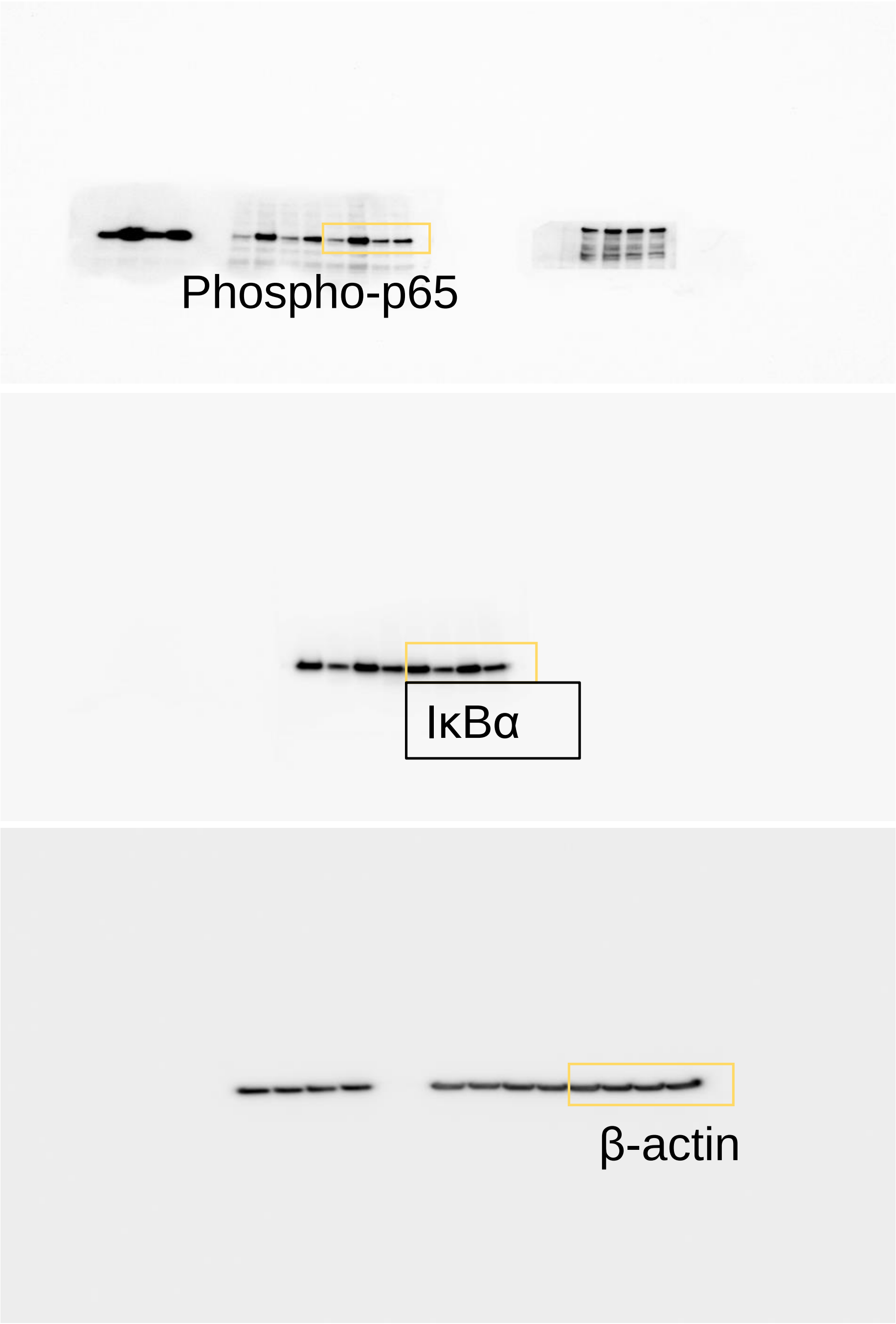

Figure S4B. SiRNA mediated knockdown of HOTAIR. HOTAIR was silenced in RAW264.7 macrophages by using HOTAIR specific SiRNA. 48h following transfection, cells were stimulated with LPS for 1h or kept untreated, harvested and protein was isolated. The protein was resolved on SDS-PAGE and immunoblotted with antibody against the indicated proteins. Figure 4D was cropped from the above images.

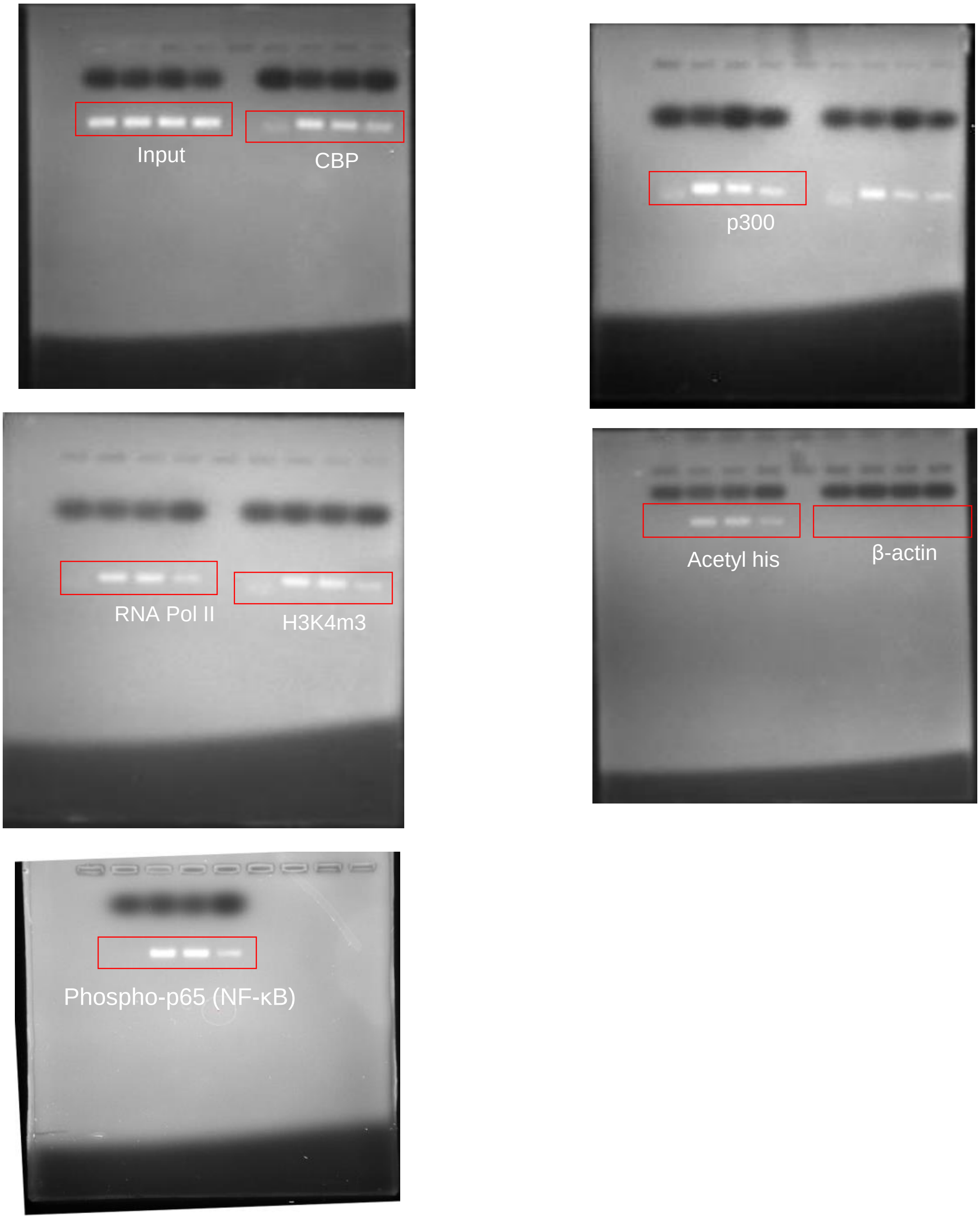

Figure S5A. Knock down of HOTAIR reduces the recruitment of transcription factors and coactivators at NF-κB binding sites on IL-6 promoter. RAW264.7 macrophage cells were transfected with HOTAIR or scramble-antisense, then treated with LPS (1.5 h). Cells were then fixed with formaldehyde and subjected to ChIP assay using antibodies specific to phospho-p65, CBP, p300, histone acetylation (Acetyl his), H3K4m3, RNA pol II and β-actin (control). The immunoprecipitated DNA fragments were analyzed by semi-quantative PCR using primers specific to the NF-κB binding regions on IL-6. Figure 6B was cropped from the above images.

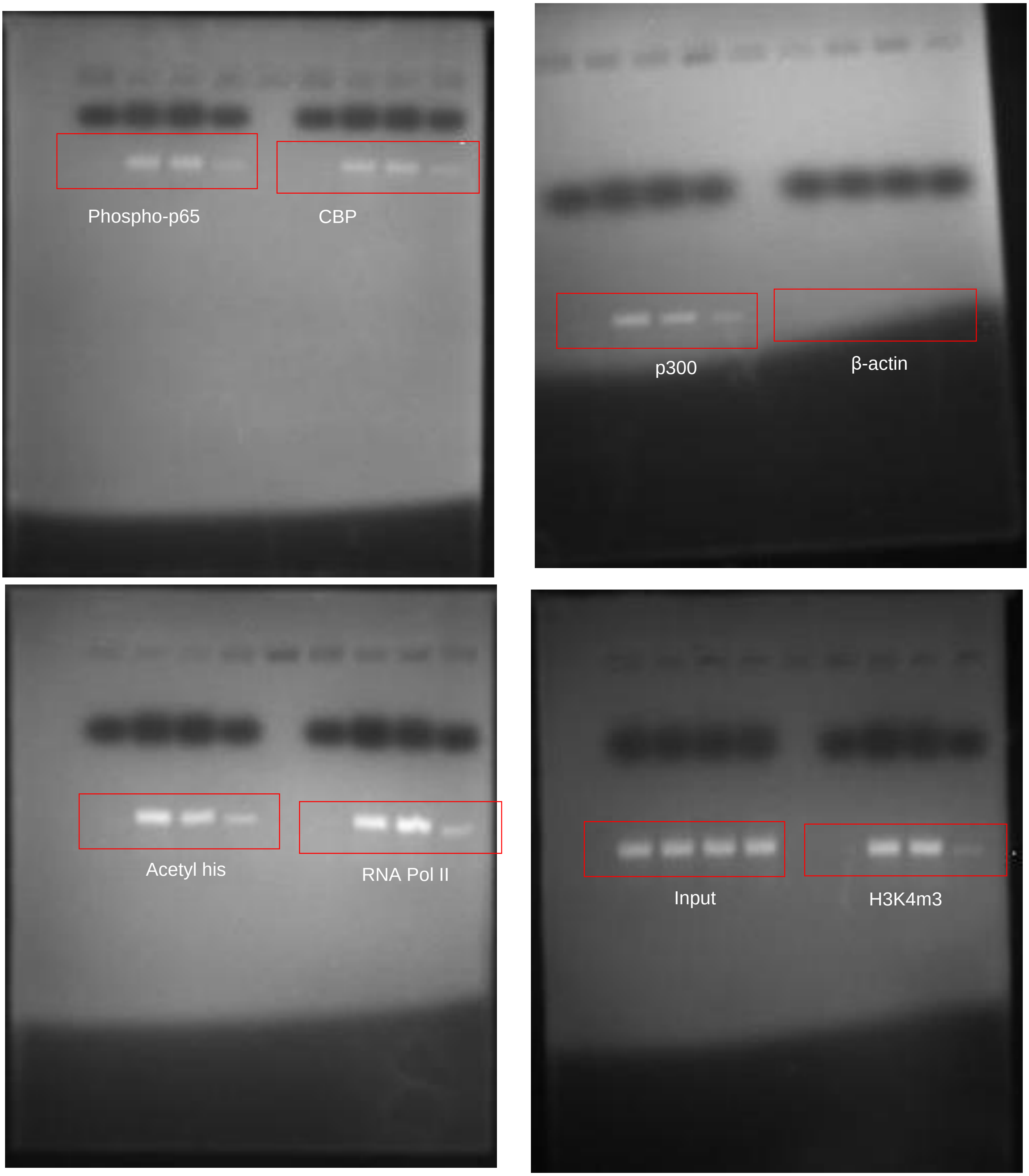

Figure S5B. Knock down of HOTAIR reduces the recruitment of transcription factors and coactivators at NF-κB binding sites on iNOS promoter. RAW264.7 macrophage cells were transfected with HOTAIR or scramble-antisense, then treated with LPS (1.5 h). Cells were then fixed with formaldehyde and subjected to ChIP assay using antibodies specific to phospho-p65, CBP, p300, histone acetylation (Acetyl his), H3K4m3, RNA pol II and β-actin (control). The immunoprecipitated DNA fragments were analyzed by semi-quantative PCR using primers specific to the NF-κB binding regions on iNOS. Figure 6D was cropped from the above images.
